# Coordination between TGF-β cellular signaling and epigenetic regulation during epithelial to mesenchymal transition

**DOI:** 10.1186/s13072-019-0256-y

**Published:** 2019-02-08

**Authors:** Congcong Lu, Simone Sidoli, Katarzyna Kulej, Karen Ross, Cathy H. Wu, Benjamin A. Garcia

**Affiliations:** 1 Epigenetics Institute, Department of Biochemistry and Biophysics, Perelman School of Medicine, University of Pennsylvania, Philadelphia, PA 19104 USA; 2 Division of Cancer Pathobiology, Children’s Hospital of Philadelphia, Philadelphia, PA 19104 USA; 3 Center for Bioinformatics and Computational Biology, Department of Computer and Information Sciences, University of Delaware, Newark, DE 19711 USA

**Keywords:** Epithelial to mesenchymal transition, Time-resolved quantitative (phospho)proteomics, Comprehensive profiling of histone modifications, Erk signaling, Histone H3 lysine 27 trimethylation, Combinatorial inhibition targeting signaling pathway and epigenetic regulator

## Abstract

**Background:**

Epithelial to mesenchymal transition (EMT) plays a crucial role in cancer propagation. It can be orchestrated by the activation of multiple signaling pathways, which have been found to be highly coordinated with many epigenetic regulators. Although the mechanism of EMT has been studied over decades, cross talk between signaling and epigenetic regulation is not fully understood.

**Results:**

Here, we present a time-resolved multi-omics strategy, which featured the identification of the correlation between protein changes (proteome), signaling pathways (phosphoproteome) and chromatin modulation (histone modifications) dynamics during TGF-β-induced EMT. Our data revealed that Erk signaling was activated in 5-min stimulation and structural proteins involved in cytoskeleton rearrangement were regulated after 1-day treatment, constituting a detailed map of systematic changes. The comprehensive profiling of histone post-translational modifications identified H3K27me3 as the most significantly up-regulated mark. We thus speculated and confirmed that a combined inhibition of Erk signaling and Ezh2 (H3K27me3 methyltransferase) was more effective in blocking EMT progress than individual inhibitions.

**Conclusions:**

In summary, our data provided a more detailed map of cross talk between signaling pathway and chromatin regulation comparing to previous EMT studies. Our findings point to a promising therapeutic strategy for EMT-related diseases by combining Erk inhibitor (singling pathway) and Ezh2 inhibitor (epigenetic regulation).

**Electronic supplementary material:**

The online version of this article (10.1186/s13072-019-0256-y) contains supplementary material, which is available to authorized users.

## Background

Epithelial to mesenchymal transition (EMT) is a reversible and plastic process in which epithelial cells lose their junctions and polarity to gain a motile mesenchymal phenotype [1]. It plays indispensable roles in physiological processes such as wound healing, but it is also involved in pathological phenotypes like cancer propagation. EMT can be orchestrated by the activation of a series of transcription factors [2], which have been found to be work together with many epigenetic regulators, including SNAI, ZEB and TWIST [3]. As a major inducer of EMT in normal mammary epithelial cells, transforming growth factor beta (TGF-β) has been shown to mediate EMT in various epithelial cells in vitro [4, 5]. Previous studies reveal that TGF-β stimulation activates multiple signaling pathways (such as the SMADs, PI3K/AKT or MAPK/ERK) and ensuing phosphorylates histone modifiers that likely affect chromatin states through the regulation of histone post-translational modifications (PTMs) expression, followed by recruitment of other histone modification binding proteins [6, 7].

Although the receptors involved in TGF-β signaling are extensively characterized [1, 8], the comprehensive profile of histone PTMs expression changes during EMT has never been investigated, lacking a global map of cross talk between cellular signaling and gene regulation driven by epigenetics. Histones are key players in epigenetics. Our laboratory has been developing novel mass spectrometry (MS)-based experimental and computational platforms to measure many facets of histone PTM biology [9–11]. Phosphorylation is the principal messenger of cellular signaling cascade. With the development of technology, MS greatly accelerates signaling research through the high-throughput quantification [12, 13]. To date, multi-omics studies offer new insights into understanding cellular processes at the molecular level [14, 15]. Therefore, a systematic quantitative analysis of histone PTMs changes in addition to the time-resolved analysis of phosphoproteome dynamics would contribute to assess how gene expression patterns cooperating with signaling pathways are modulated upon EMT reprogramming.

In this study, we present a time-resolved multi-proteomics strategy to monitor EMT development, including the quantitative analysis at proteome, phosphoproteome and histone PTMs level, respectively. By taking the advantages of SILAC (stable isotope labeling by amino acids in cell culture)-based quantification, proteins concentrated at areas of cell–cell adhesion and cell migration were grouped after 1-day TGF-β stimulation, suggesting overall that structural proteins rearrangements evoked in the later transition state. Significant regulations of phosphorylations were detected after only 5-min treatment, leading to the prediction that Erk signaling was activated upon immediate stimulation. Meanwhile, a monotonic increase in H3K27me3 (histone H3 lysine 27 trimethylation) was observed over the time-course using label-free quantification methodology. This PTM is catalyzed by Ezh2, which is itself regulated by phosphorylations. The combined analysis revealed a potential mechanism for Ezh2 activation and up-regulation of H3K27me3 during EMT. We thus hypothesized that a combined inhibition of signaling pathways Erk and epigenetic regulator Ezh2 would have a pronounced effect in preventing EMT reprogramming and validated our conjecture through the influence on cell phenotypic and histone PTMs expression changes derived from various inhibitors.

## Results

### Experimental design to monitor EMT development

**Fig. 1:**
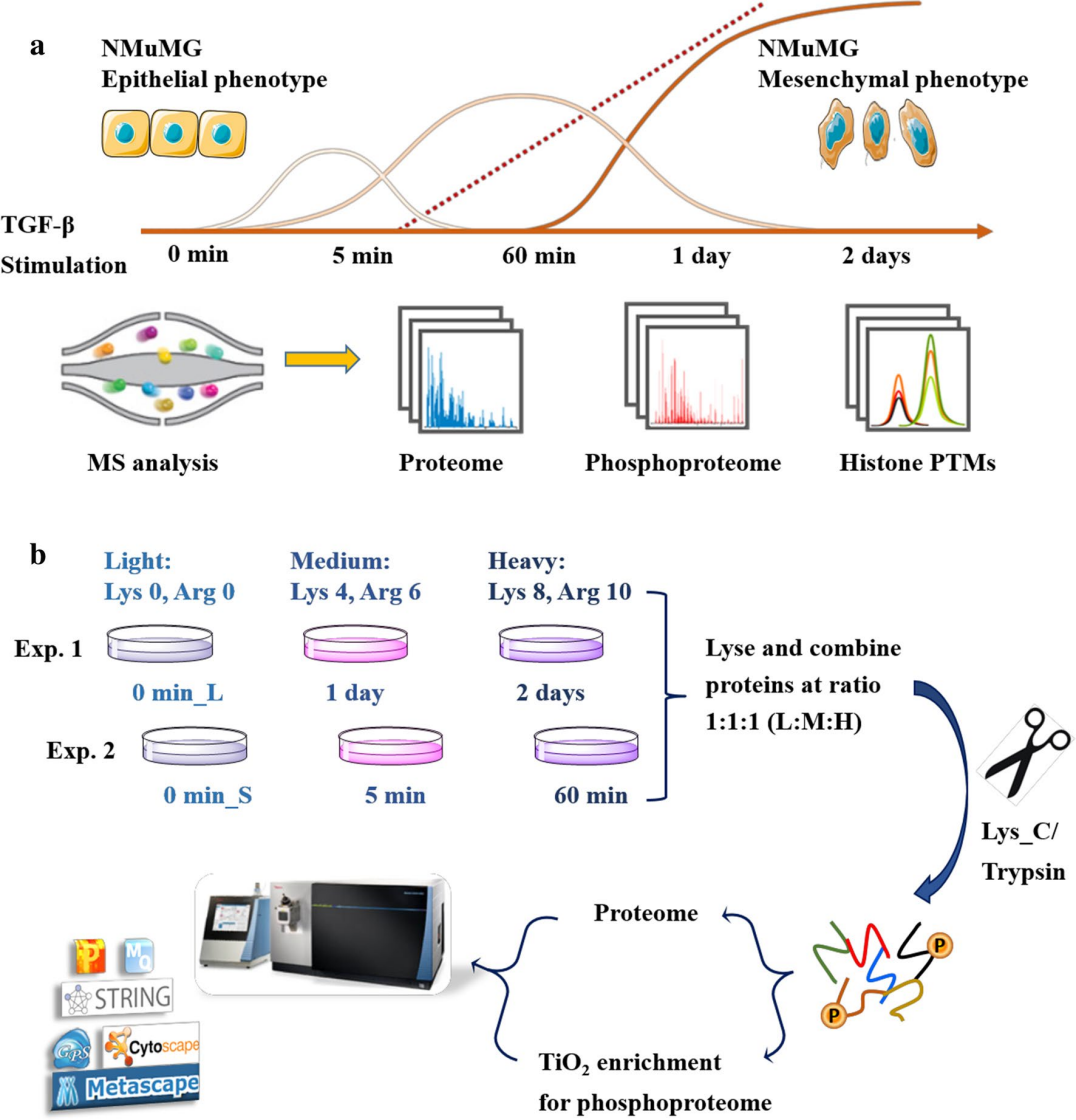
Experimental design for study of cross talk between TGF-β cellular signaling and epigenetic regulation during EMT. **a** TGF-β was added to induce EMT in NMuMG cells, and expressions at proteome, phosphoproteome and histone PTMs level were quantified, respectively. The different curves implied distinct regulation patterns during EMT programming. **b** Workflow for quantitative and time-resolved analysis of (phospho)proteomes using triple SILAC labeling. Two experiments with biological triplicates were performed with 0 min as a common time point

Characterizing EMT is an important research field, as it plays indispensable roles in cancer propagation. Although the TGF-β signaling, which can trigger EMT when the pathway is activated, is extensively characterized, the coordination between cellular signaling and epigenetic regulation needs further studies. Here, we applied our in-house developed multi-omics strategy to monitor proteome, phosphoproteome and histone PTMs expression changes in a cell model undergoing EMT (Fig. 1a). Simply, we used NMuMG (normal murine mammary gland epithelial) cells exposed to TGF-β, which is an established model to investigate EMT in cell culture [16, 17]. Cells were collected after different stimulation times, from 5 min to 2 days. Proteome, phosphoproteome and histone PTMs were further quantified by MS to investigate the correlation between protein changes, signaling pathways and chromatin modulation dynamics during EMT.

Quantitative analysis of global proteome and phosphoproteome at five time points during EMT programming was achieved by adopting metabolic labeling (Fig. 1b). Specifically, three populations of NMuMG cells were SILAC encoded with both arginine and lysine using three distinct isotope forms (K0R0, K4R6 and K8R10) and stimulated by TGF-β (5 ng/ml). Two time-course experiments were combined using the common time point 0 min (no stimulation), providing a five-time-point profile to check immediate early (5 min), early (60 min), late (1 day) and very late (2 days) (phospho)proteome response to TGF-β. Phosphopeptides were enriched using TiO_2_ beads. Proteome and phosphoproteome were detected by Orbitrap Fusion and further analyzed through various bioinformatics tools.

### TGF-β stimulation successfully induced EMT

**Fig. 2:**
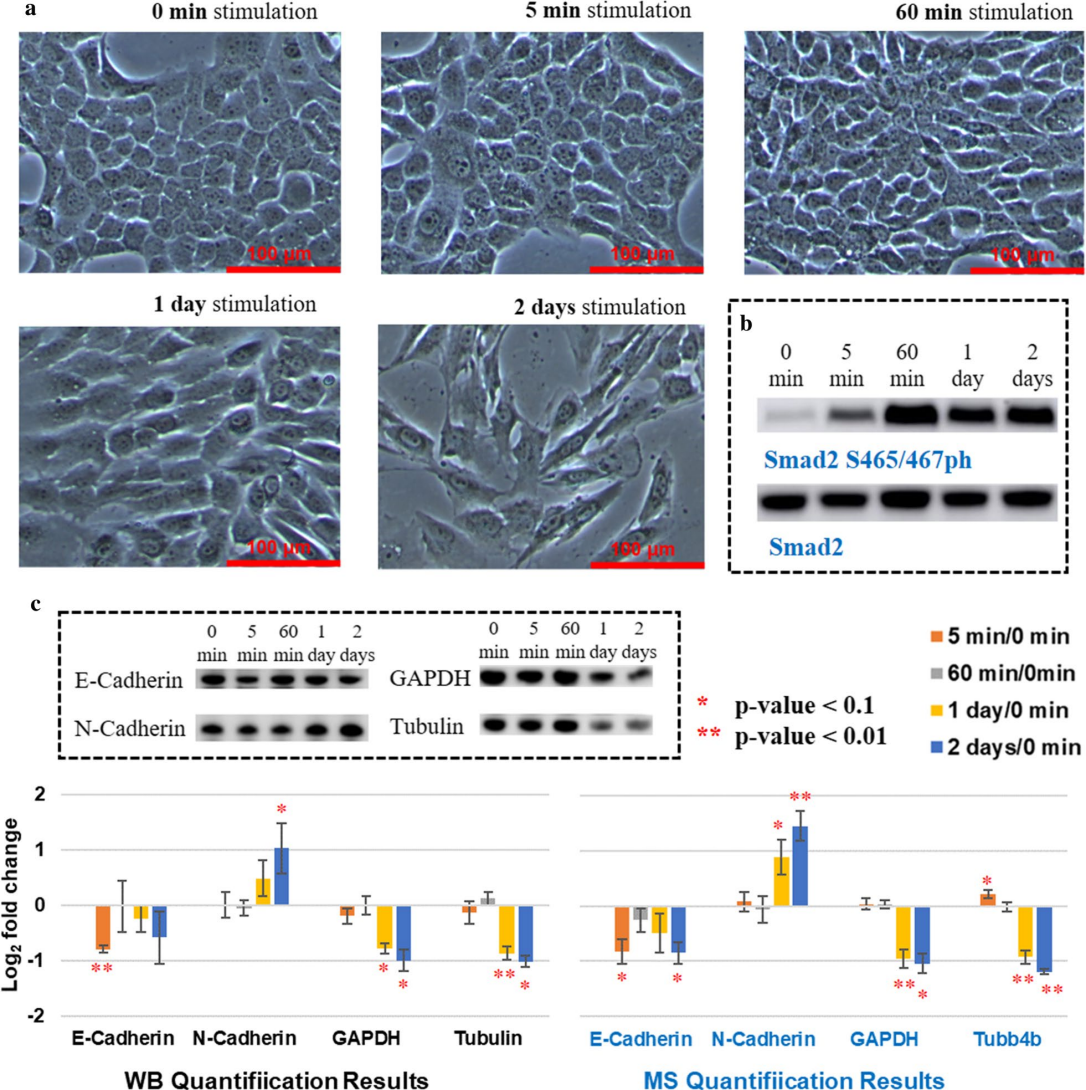
Changes of cell morphology and expression of given proteins after adding TGF-β. **a** Cells show fibroblast-like features over the stimulation time-course, indicating a mesenchymal phenotype switch. **b** WB results indicate that Smad2 was phosphorylated in 5 min after adding TGF-β, which is a direct target of TGF-β signaling and should be a nice positive control. **c** Expression changes of given proteins quantified by WB and MS, respectively. Data are means ± SD (standard deviation) of triplicates. As expected, epithelial marker protein E-cadherin and mesenchymal marker protein N-cadherin were down-regulated and up-regulated, respectively

First, we evaluated whether EMT happened or not after TGF-β treatment. According to the literature, the phenotypic changes that occur during EMT involve the disassembly of cell–cell contact, separation of cells and remodeling of cytoskeleton, which generate fibroblast-like cells that express mesenchymal markers [18]. After 2-day treatment, dramatic morphological changes were observed (Fig. 2a); cells became larger than untreated ones (0 min), and the shape of cells changed from polygon into fusiform, showing features of mesenchymal cells.

Phosphorylation of Smad2 at S465 and S467 is a direct target of TGF-β signaling [4], which should be a nice positive control to prove that the pathway was activated. Unfortunately, it was not possible to quantify the Smad2 pS465/pS467 changes by MS because it locates at the C-term tail of Smad2 and there is no lysine or arginine that can be isotope-labeled (amino acids sequence from 452 to 467: VLTQMGSPSVRCSSMS). Thus, we checked the Smad2 phosphorylation changes by western blotting (WB), where clearly pinpointed that Smad2 was phosphorylated right after adding TGF-β (5 min) and the phosphorylation state remained during EMT programming (Fig. 2b), suggesting the activation of Smad signaling and presumable EMT reprogramming.

Besides the phenotype remodeling and phosphorylation of target proteins of signaling, major changes which occur during EMT are the down-regulation of epithelial markers expression (such as E-cadherin) and the acquisition of mesenchymal markers abundance (such as N-cadherin). To further confirm the mesenchymal transition, we quantified the expression changes of E-cadherin and N-cadherin by WB, as well as commonly used loading control proteins, GAPDH and beta-tubulin (Fig. 2c, left). E-cadherin was down-regulated and N-cadherin up-regulated after stimulation as we expected. GAPDH is a protein that is commonly used for normalization in many studies; however, the expression of this protein may change with hypoxia [19] which makes it inappropriate as a control when working on hypoxic samples, like EMT studies. Because EMT is a process which involves the structural organization in the cells, it would be not be appropriate to use structural proteins such as tubulin as control. Thus, it is not surprising to see that GAPDH and tubulin were significantly changed in the late transition. Similar quantification results were obtained through MS (Fig. 2c, right) with an average linear correlation *R*^2^ > 0.94 with WB data (Additional file 1: Figure S1), demonstrating the accuracy of our MS-based proteome-wide quantification.

Combining all phenotype, marker protein and phosphorylation expression changes, it can be concluded that TGF-β successfully activated signaling pathways and subsequently induced EMT in NMuMG cells.

### (Phospho)proteome expression changes during EMT

The distributions of raw protein and phosphosite intensities were firstly explored through boxplots, where similar distributions before and after data normalization were presented (Additional file 1: Figure S2), implying that the different conditions were properly mixed and injected into MS. The analysis of the proteome resulted in the total quantification of 2977 proteins, of which 2328 (78.2%) were quantified across all conditions (Additional file 1: Figure S3A and Additional file 2: Table S1). 3131 (50.3%) out of 6230 phosphosites were measured in all conditions (Additional file 1: Figure S3A and Additional file 3: Table S2A). Only 854 proteins were identified in both proteome and phosphoproteome datasets (Additional file 1: Figure S3B). This was expected, largely stemming from the low abundance and high complexity of phosphoproteome. However, it was also a limitation, because only the overlapped dataset of phosphorylations can be corrected by the protein abundance to assess real regulations of the phosphorylation events. Meanwhile, high phosphoproteome coverage was facilitated by high enrichment specificity (~ 90% of identified peptides were phosphorylated in each experiment) (Additional file 1: Figure S3C), indicating the good data quality and reproducibility.

**Fig. 3:**
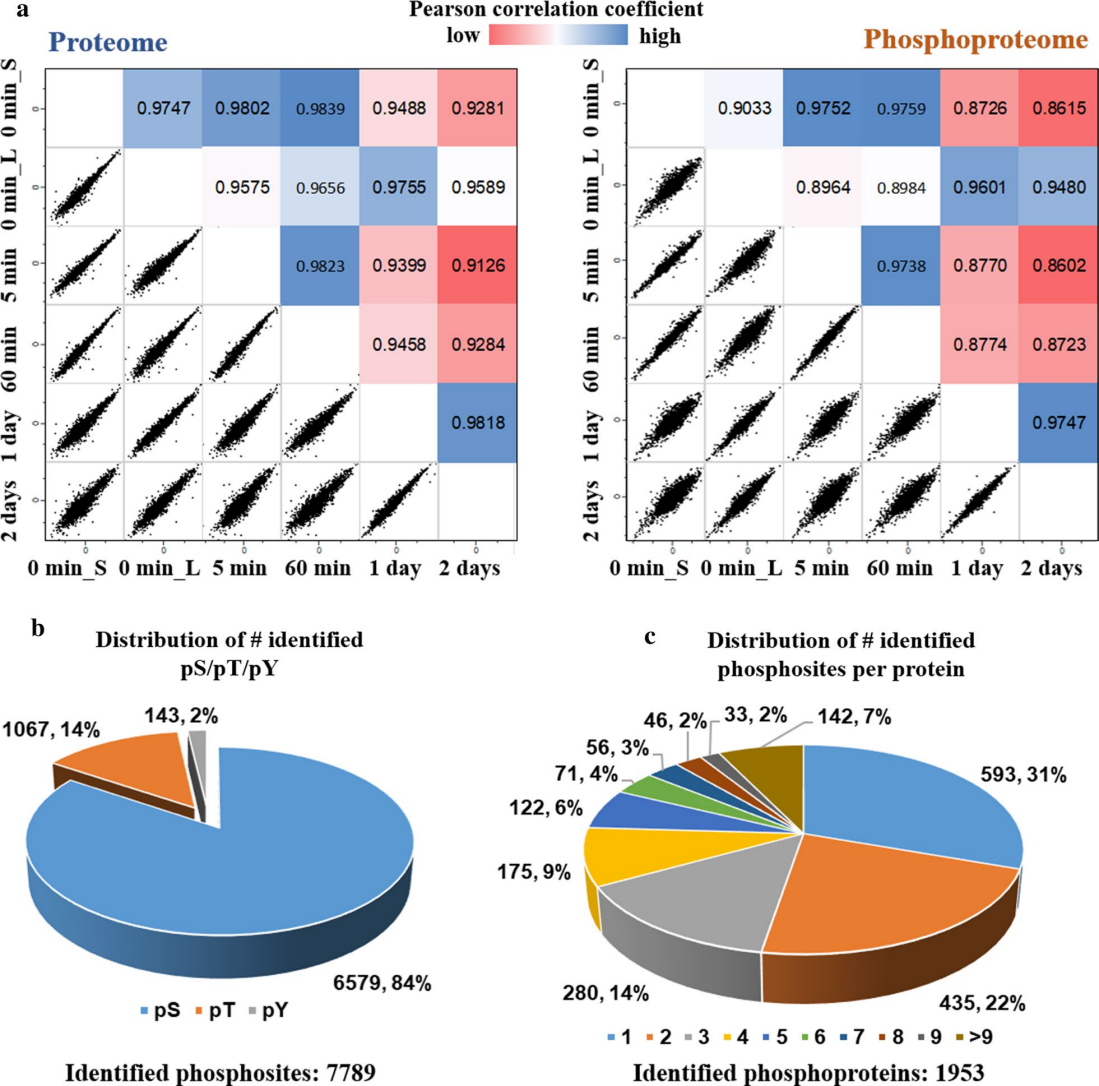
Quantitative analysis of proteomes and phosphoproteomes. **a** Scatter plots and person correlation coefficient *R* representing the correlation of (phospho)proteome between indicated times after TGF-β stimulation. 0 min_L indicates the sample of 0 min from Exp. 1 and 0 min_S from Exp. 2 as shown in Fig. 1b. **b** Pie chart showing the distribution of the number of identified phospho-serine (pS), phospho-threonine (pT) and phospho-tyrosine (pY). **c** Pie chart showing the distribution of the number of identified phosphosites per protein. Around 69% proteins were identified with more than one phosphosite

We then performed Pearson correlation coefficient analysis for whole proteome and phosphoproteome based on the normalized intensities to assess the similarity between different conditions (Fig. 3a). It is reasonable to find that conditions mixed and detected within the same MS run, namely Exp.1: 0 min_L, 1 day and 2 days; Exp. 2: 0 min_S, 5 min and 60 min (Fig. 1b), have higher correlations due to the technical and instrument variation. A high correlation among 0 min_S, 5 min and 60 min at proteome level was observed with *R*^2^ values higher than 0.98. Proteomes after 1 day or 2 days stimulation were observed to be less correlated to 0 min_L, indicating clear differences after longer TGF-β treatment at proteome level. Compared to proteome results, phosphoproteome has smaller *R*^2^ values, respectively, implying a large variety of phosphorylation regulation. Same as proteome, phosphoproteome after 2-day stimulation was also found to be the most different compared to 0 min l, suggesting the biological variation increased along with TGF-β treatment time.

The normal distribution of serine (S): threonine (T): tyrosine (Y) phosphorylation frequencies on cellular proteins is about 80:20:1 in most mammalian systems [13]. Our phosphoproteomics results obtained a ratio of 84:14:2 (Fig. 3b), highlighting that our results are in line with expectations. More than 67% of phosphosites were identified with a localization confidence higher than 0.75 (Additional file 1: Figure S3D) with an average localization probability 0.95, indicating that most of our identified phosphorylations are accurately mapped at single amino acid (S, T, Y) resolution. Phosphosites were determined on a wide variety of proteins, and around 69% proteins were defined with more than one phosphosite (Fig. 3c). For instance, Prelamin-A/C, a proved protein involved in the well-known PI3K/AKT signaling activated by TGF-β [20], was detected with 33 phosphosites which may be regulated differently across time-course and have different corresponding functions.

### Time-resolved phosphoproteome dynamics during EMT

TGF-β signaling begins with activation of TGF receptors and extends through numerous signaling pathways, such as the SMADs, PI3K/AKT, or MAPK/ERK pathways [4]. Subsequently, these activated pathways mediate the phosphorylation of a large number of substrate proteins and cross talk with each other at multiple levels, leading to gene expression albeit regulated by post-transcriptional and post-translational mechanisms. These events are separated in time, and a time-resolved analysis of phosphoproteome dynamics is critical to understand cellular signaling during EMT development.

**Fig. 4:**
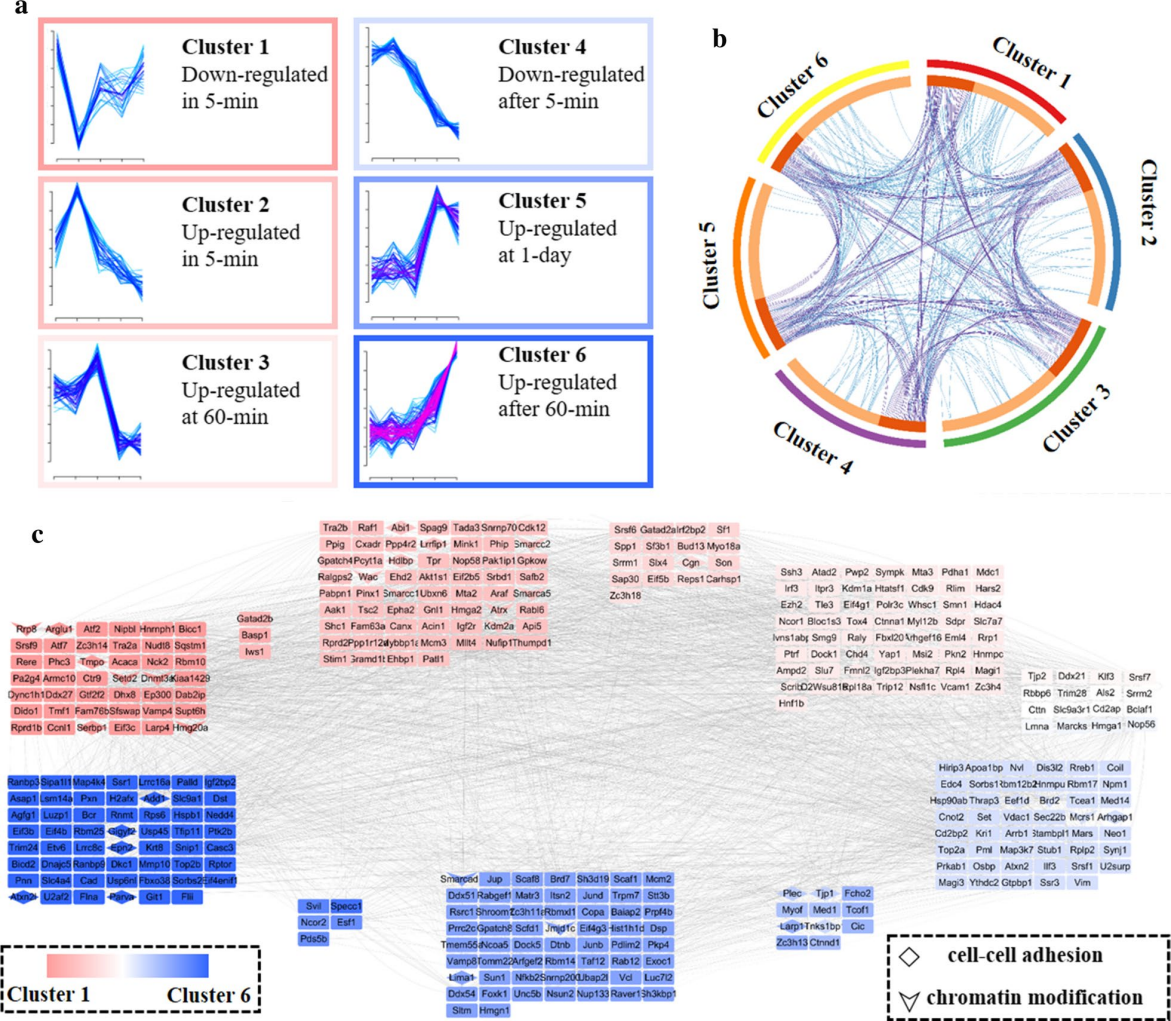
Time-resolved analysis of the phosphoproteome during EMT. **a** Cluster profiles for phosphoproteome. Phosphosites with at least four valid numbers out of five time points and ANOVA test smaller than 0.05 were clustered in abundance using fuzzy c-means. **b** Circos plots showing the overlaps of phosphoproteins between 6 distinct clusters. **c** Phosphorylation events ordered on the time points where phosphorylation peaked by averaging the clusters numbers

Significantly regulated phosphosites or proteins quantified in at least four out of five time points with ANOVA *p* value < 0.05 were classified through fuzzy c-means clustering analysis [21]. This allowed for the grouping of 888 phosphosites and 1180 proteins into six clusters (Fig. 4a and Additional file 1: S4B). As mentioned, 69% proteins were modified with more than one phosphosite (Fig. 3c). We then investigated whether different phosphosites (only those used for clustering) within the same protein were regulated in the same manner. Interesting, 78 out of 511 phosphoproteins (15.2% of phosphoproteins used for clustering) that had a regulated phosphorylation event also had at least one more event with different trend. The circus plot in Fig. 4b shows the overlaps between phosphoproteins in different clusters, clearly reflecting that proteins could contain different phosphorylation events. For instance, Trim28 (transcription intermediary factor 1-beta) carried four serine phosphosites, exhibiting in cluster 2 (S26ph), cluster 3 (S23ph), cluster 4 (S21ph) and cluster 5 (S473ph), respectively. Previous study reveals that Trim28 contributes to EMT via regulation of E-cadherin and N-cadherin expression through histone modifications [22]. We also detected three serine phosphosites on Vim (Vimentin), classified in cluster 1 (S430ph), cluster 5 (S419ph) and cluster 6 (S214ph), respectively. Tjp1 (Tight junction protein ZO-1) was determined with two serine phosphosites (S166ph and S168ph) belonging to cluster 5, one serine phosphosites (S912ph) in cluster 4 and serine phosphosites (S178ph) in cluster 3. Vim is usually found in various non-epithelial cells, especially mesenchymal cells. Tjp1 regulates cell adhesion and matrix remodeling and can be used as an epithelial marker [4]. These different phosphorylation expression patterns within the same protein imply that phosphorylation events on proteins can have very different regulation, complicating the understanding of the functions of phosphorylations on a given protein.

In order to get a better understanding of protein–protein interactions as well as regulated protein functions at different stimulation time points, we then ordered the phosphoproteome events based on the time point where phosphorylation peaked by averaging the clusters numbers (Fig. 4c). Protein–protein interaction analysis generated through String [23] showed that all regulated phosphoproteins were highly interacted with each other. GO-BP (gene ontology biological processes) enrichment was further performed to determine the biological enrichment represented in each cluster (Additional file 1: Figure S4C and Additional file 3: Table S2D). Phosphorylation changes classified in cluster 1 and cluster 2 were significantly concentrated at the area of mRNA metabolic process, implying that signaling cascade was activated immediately (in 5 min) to mediate gene expression. Long-term phosphorylation response (> 1 day) was grouped associated with actin cytoskeleton organization, overall suggesting that the stimulation remodeled structural proteins involved in cell adhesion in the later transition state.

Previous studies of EMT focused on investigations at later transition states, e.g., from 1 to 8 days [17, 24], and thus, they could miss the important quick signaling response, like after 5-min stimulation. So, we further analyzed phosphoproteins significantly regulated in the immediate early response. Independently from their phosphorylation status changes, these proteins were grouped into two different clusters (Cluster 1 and 2). Further enrichment analysis showed that the majority of phosphoproteins are located in nucleus and specifically related with RNA splicing (Additional file 1: Figure S5), an important element of transcriptional regulation [25]. It is noteworthy to mention that parts of phosphoproteins in cluster 2 (up-regulated in 5 min) belong to the SWI/SNF family. This superfamily has been well characterized with respect to chromatin remodeling processes and plays critical roles in differentiation and proliferation [26].

### Phosphosite-kinase specific analysis

**Fig. 5:**
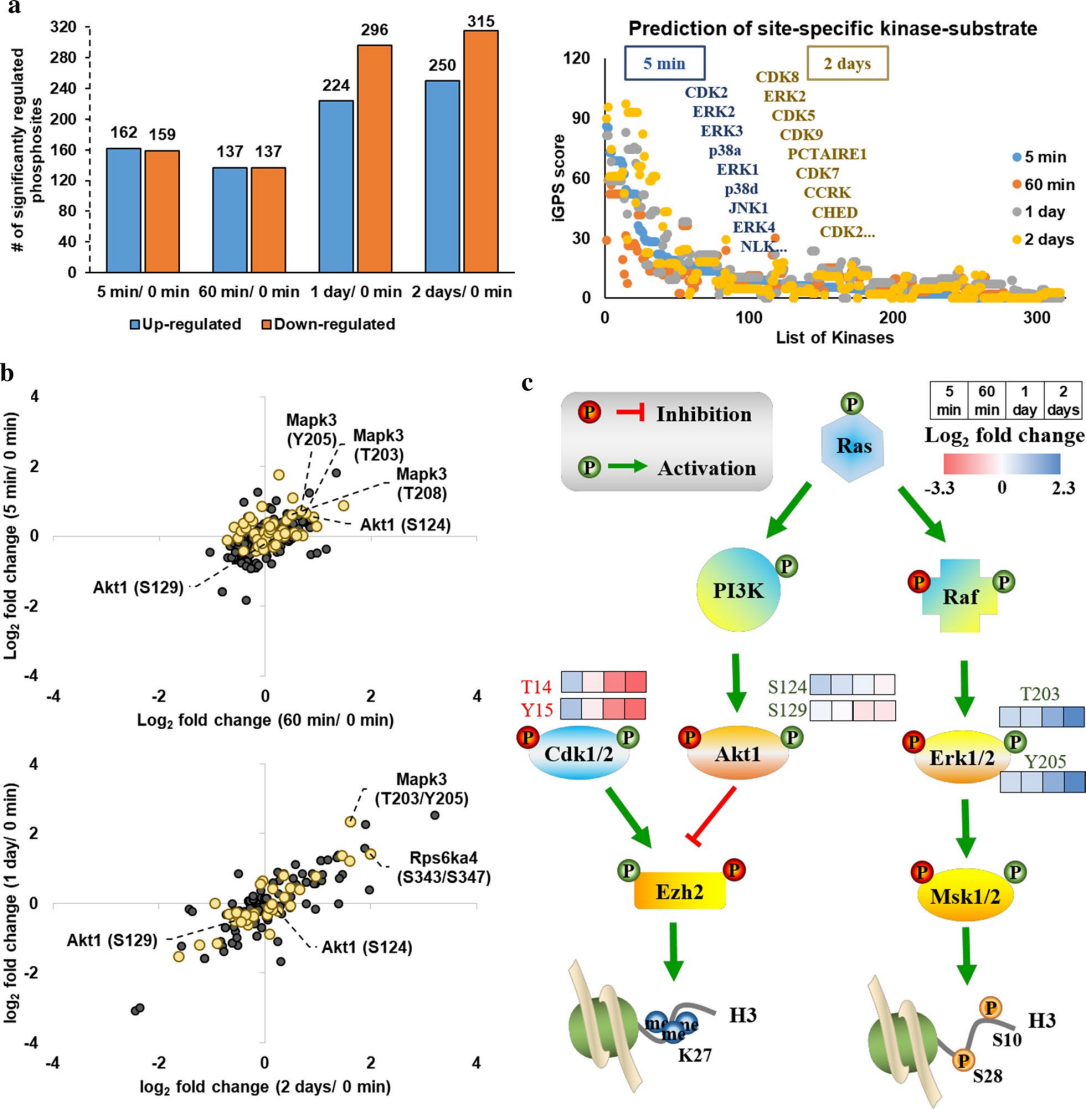
Phosphosite specific analysis. **a** iGPS prediction of kinases responsible for significantly regulated (*p* value < 0.05 based on triplicates) phosphopeptides at the indicated times after TGF-β stimulation. Top 9 kinases predicted for 5-min and 2-day regulation (compared to 0 min) were labeled in scatter plot (right). Histogram (left) showed the numbers of significantly regulated phosphosites at given stimulation times. **b** Scatter plots of log_2_ fold change of 5 min/0 min versus 60 min/0 min (up) and 1 day/0 min versus 2 days/0 min (bottom) for phosphorylation expression of kinases detected in our dataset. Kinases highlighted in yellow have been previously described with histone modifiers. **c** A cartoon representing signaling pathways related to histone modifications. Regulated phosphosites, which are associated with enzymatic activity, were shown with varying hues indicating the log_2_ fold change of relative abundance compared to 0 min. Mapk3, also named as Erk1. Rps6ka4, also named as Msk2

In eukaryotes, hundreds of kinases specifically modify ~ 30% of all cellular proteins to orchestrate numerous biological processes such as signal transduction [13]. Identification of protein phosphosites with their cognate kinases is a key step to delineate molecular dynamics and cellular processes. Therefore, the site-specific kinase-substrate relations prediction software iGPS [27] was utilized to construct a kinase-substrate phosphorylation network for these significantly regulated phosphosites (*p* value < 0.05 based on biological triplicates) at different time points (Fig. 5a and Additional file 3: Table S2B). Listed kinases in the scatter plot were predicted kinases containing highest iGPS scores for 5-min and 2-day mediation, respectively. Phosphosites significantly regulated after 5-min stimulation were linked to ERK, p38 or JNK, while long-term regulation (2 day) was governed by CDK family. ERK/AKT pathway is required for TGF-β-induced EMT in vitro through the regulation of EZH2 (histone-lysine *N*-methyltransferase EZH2) expression [7]. Activation of p38 or JNK also mediates EMT and is critical for down-regulation of E-cadherin [17]. It is exciting to find that these kinases were activated as early as in 5 min of TGF-β stimulation since previous studies mainly investigate long-term events of EMT [17, 24]. These observations emphasized the importance of ERK signaling early response to TGF-β in EMT programming.

Most protein kinases are dephosphorylated and inactive in the basal state, and they are themselves controlled by phosphorylation, either by other kinases or by other copies of themselves (autophosphorylation). Then, the phosphoproteome dataset was mapped to the known kinome (Additional file 1: Figure S6), where 113 kinases were detected with 363 phosphosites. Kinase phosphorylation expression changes calculated in log_2_ fold change to unstimulated condition were plotted out to evaluate the correlation between individual stimulation times (Fig. 5b). The early stimulation time points (5 min and 60 min) did not maintain a visible correlation, showing a random pattern of changes in abundance. To the opposite, a sustained increase or decrease in phosphorylation expression on kinases was observed between day 1 and day 2, showing a linear correlation after day-scale stimulation. The correlation between kinase phosphorylation express suggests that kinases involved in early activation of EMT signaling and late remodeling of cytoskeleton had multiple phosphosites and different regulation patterns.

Regarding that we are mainly interested in the regulatory networks implicated in modeling cell phenotype, kinases that have been reported with histone modifiers and might be involved in epigenetics regulation are highlighted in Fig. 5b. Among these kinases, Mapk3 (also named as Erk1), Akt1 and Cdk1/2 were the most attractive with respect to histone modifications (Fig. 5c). Dual phosphorylation of T203 and Y205 causes dramatic conformational change of Erk1, which enable full enzyme activation [28] and interaction of Erk1 with its substrates, including Msk1/2. Recent evidence has shown that Msk1/2 can directly phosphorylate histone H3 at the sites S10 and potentially site S28 [29, 30]. On the other hand, Akt1 and Cdk1/2 can mediate Ezh2 activity, which is a well-known H3K27me3 writer. To verify whether histone modifications, especially H3 PTMs, were regulated in accordance with the observed kinase phosphorylations, we performed a comprehensive histone PTM analysis using our in-house developed label-free quantification workflow.

### Comprehensive profiling of histone modification changes

**Fig. 6:**
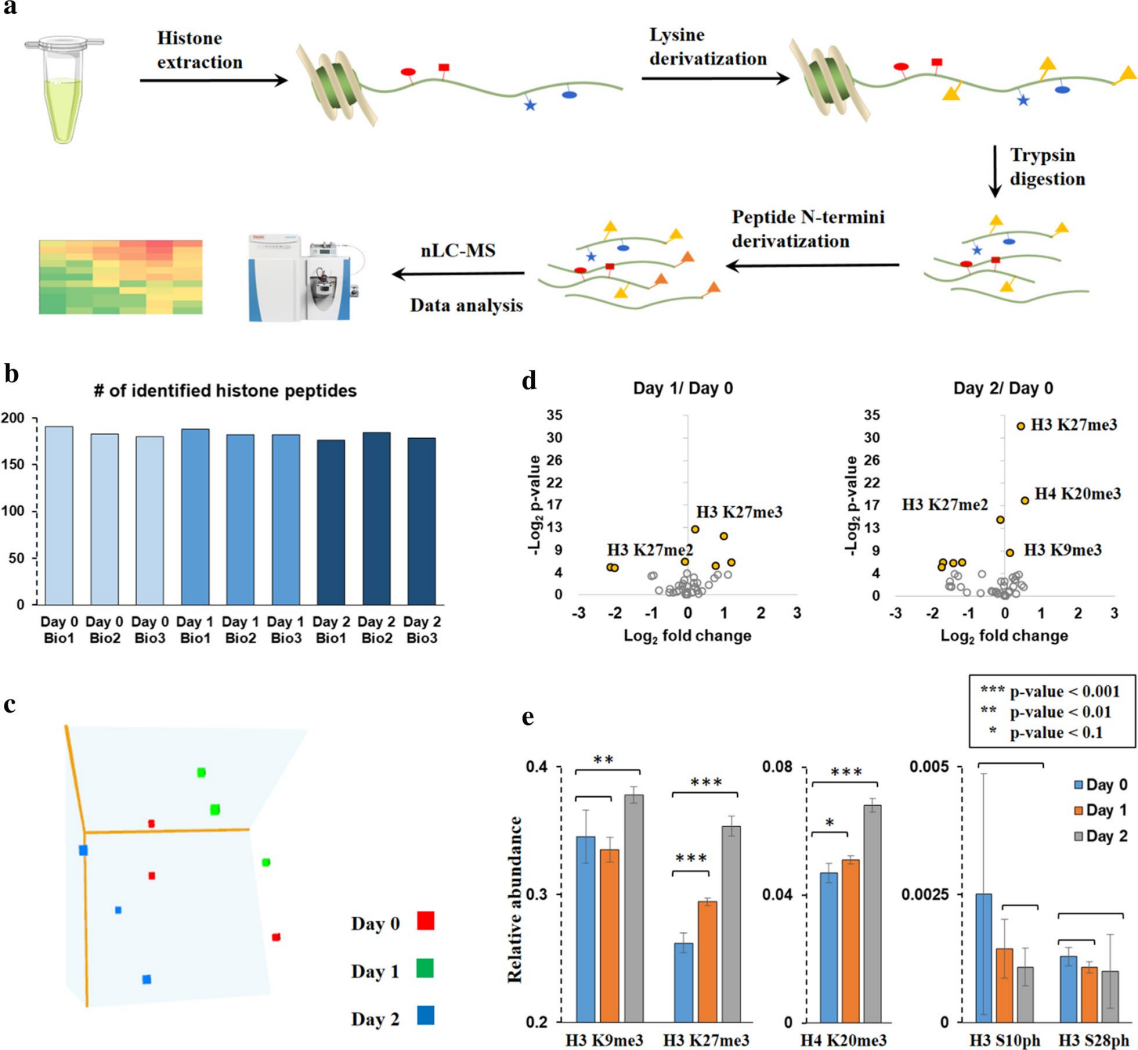
Comprehensive histone PTM analysis during EMT. **a** Workflow for label-free quantification of histone PTMs. **b** Number of identified histone peptides carrying different types of modifications at indicated TGF-β stimulation times with biological triplicates. **c** Three-dimensional PCA (principal component analysis) representing histone PTM types was clustered separately based on different stimulation times. The data supplied to generate PCA were all detected histone peptides provided in Table S3A, including modified and unmodified peptides. **d** Volcano plot presenting fold change and significance of histone H3 and H4 single PTMs relative abundance. Yellow bubbles are significantly regulated marks, i.e., with a *t* test *p* value < 0.05 or > 4.32 when − log2 transformed. **e** Relative abundance changes of selected single histone PTMs. Error bars represent standard deviation (*n* = 3)

The global profiling of histone PTM changes was obtained by using a label-free quantification workflow established by our laboratory (Fig. 6a) [31]. Cells were treated with TGF-β for 0 day, 1 day and 2 days before harvesting. The reason why we did not include 5 min and 60 min is that from our previous studies on histone dynamics, histone modification changes have slower dynamic changes than phosphorylations, normally on a scale between hours to days [32]. Histones were purified via acidic extraction and then chemically derivatized with propionic anhydride to block free lysine residues. After trypsin digestion, a second round of propionylation was performed to derivatize peptides N-termini. Samples were analyzed by nLC-MS/MS through data-independent acquisition (DIA) method, and peptide quantification was extracted by our in-house developed software EpiProfile 2.0 [33].

Around 200 different histone peptides carrying different kinds of modification types were quantified (Fig. 6b and Additional file 4: Table S3A), including peptides from histones H3, H4, H2A, H2B and H1 and their variants. For instance, there are more than seven known sequence variants of histone H3 in mammalian cells [34]. These H3 variants have highly conserved sequences differing only by a few amino acids. H3.1, H3.2 and H3.3 are the most abundant H3 variants. By using bottom-up MS strategy, H3.1/H3.2 can be distinguished from H3.3 by the identification of peptide 27–44 residues as residue 31 in H3.1/H3.2 differs from H3.3. Without other mentions, all modifications discussed in the area 27–44 were detected on H3.1/H3.2.

PCA (principal component analysis) showed that triplicates of the same condition clustered together and different conditions grouped separately (Fig. 6c), indicating the histone PTMs variances over the TGF-β treatment. A heatmap including the overall abundance of each single mark was generated to inspect the most abundant modifications of histone H3 and H4 (Additional file 1: Figure S7A and Additional file 4: Table S3B), where H3K9me2/me3, H3K27me2/me3 and H4K20me2 were found to be the dominant marks. For instance, relative abundances of all modified forms of the peptide carrying given modification (K27me3 + K27me3K36me1 + K27me3K36me2…) were summed up to estimate the global relative abundance H3K27me3. Then, PTMs relative abundance was organized in hierarchical clustering to see the trends during EMT (Additional file 1: Figure S7B), creating an overall dynamics of histone marks. Volcano plots presenting fold change and significance for H3 and H4 single marks (Fig. 6d) demonstrated that H3K27me3 was the most significant up-regulated histone mark.

Previous study suggests that H3K27me3 is required for EMT [35], and it is in accordance with our histone PTMs quantification profile where a monotonic increase in H3K27me3 was found during EMT (Fig. 6e). H3K27me3, catalyzed by Ezh2, is a well-characterized histone modiFIcation associated with gene silencing. The activity of Ezh2 is regulated by phosphorylation [36, 37]. Unfortunately, we were not able to quantify Ezh2 phosphorylation changes due to its low abundance even after enrichment. However, we identified activity-related phosphosites within kinases which can regulate Ezh2 activity (Fig. 5c). According to studies, Akt1 and Cdk1/2 can mediate recruitment of Ezh2 to maintain H3K27me3 level through different phosphosites with opposite effects. Akt1 phosphorylates Ezh2 at S21 and suppresses its methyltransferase activity [36], while Cdk1/2 phosphorylates Ezh2 at T350 which is important for recruitment of Ezh2 to H3K27 [37]. From our phosphoproteome dataset, S124ph and S129ph of Akt1, which are required to activate Akt1 [38], were found to be decreased during EMT. In the meanwhile, T14ph and Y15ph of Cdk1, which can inhibit Cdk1 enzymatic activity [39], were reduced along with transition. The combined analysis revealed a potential mechanism for Ezh2 activation and up-regulation of H3K27me3 during EMT through the deactivation of Akt1 and loss of suppression of Cdk1.

Erk1 was activated during EMT due to the expression of T203ph/Y205ph (Fig. 5c). Msk1/2, as Erk1 substrates, is known to directly phosphorylate histone H3 at the sites S10 and S28 and may further induce a methylation–acetylation switch of the adjacent residues (K9 and K27) [29, 30], while we did not observe a significant regulation of its target H3 S10ph or S28ph (Fig. 6e). It can be potentially explained by that the binding protein complexes of adjacent residues K9me3 or K27me3 may suppress the recruitment of binding proteins for S10 or S28ph as we found a dramatic increase in nearby H3K9me3 or K27me3 after stimulation.

Nevertheless, our data highlight that histone PTMs are dynamically affected during EMT, in particular the gene silencing mark H3K27me3. The mechanism of H3K27me3 expression might be regulated by Erk1, Cdk1/2 and Akt1. Considering the high complexity and cross talk of EMT regulatory network, we speculate that a combinatorial inhibition targeting EMT transcription factor signaling and epigenetic mechanisms would be a seductive approach noteworthy to test. We then performed a phenotypic assay to verify the ability of an epigenetic inhibitor and/or kinase inhibitor in blocking EMT.

### Combination of inhibitor treatment affects EMT process

Different inhibitors were tested either alone or in combination with each other to check their effect on cell phenotypic alterations (Additional file 1: Figure S8A). Specifically, LDN-214117 was introduced to block Alk2, which is a TGF-β receptor, acting as a positive control. DMSO was used as a negative control. Inhibitors for Cdk7 (BS-181HCl), Cdk family (PHA-793887) and Erk (AZD6244) were selected based on phosphosite-specific analysis results (Fig. 5a) as well as indicated by previous reports [16]. Ezh2 inhibitors UNC1999 and GSK126 were utilized to alter H3K27me3 expression. Cells were pre-treated with inhibitors for 1 day before providing TGF-β stimulation for two more days with the attendance of indicated inhibitors.

To summarize the cell phenotype switch results after 3-day individual treatments (Additional file 1: Figure S8A), Ezh2 inhibition alone did not block the transition process, showing a similar phenotypical change as the DMSO control with mesenchymal feathers. The kinase inhibitors targeting Erk had a better disruption influence on EMT processing than blocking Cdk family or Cdk7, either alone or in concurrent with Ezh2 inhibitors. Even Erk inhibitor achieved the most efficient EMT repression among mono-inhibitions, cells manifested abnormal shape with a fuzzy cell outline. This side effect was resolved by a combination treatment plus Ezh2 inhibitor. Almost all cells upon the combination inhibition of Erk and Ezh2 retained epithelial signatures along with normal sharp outline, suggesting a potential effective strategy to prevent mesenchymal transition.

**Fig. 7:**
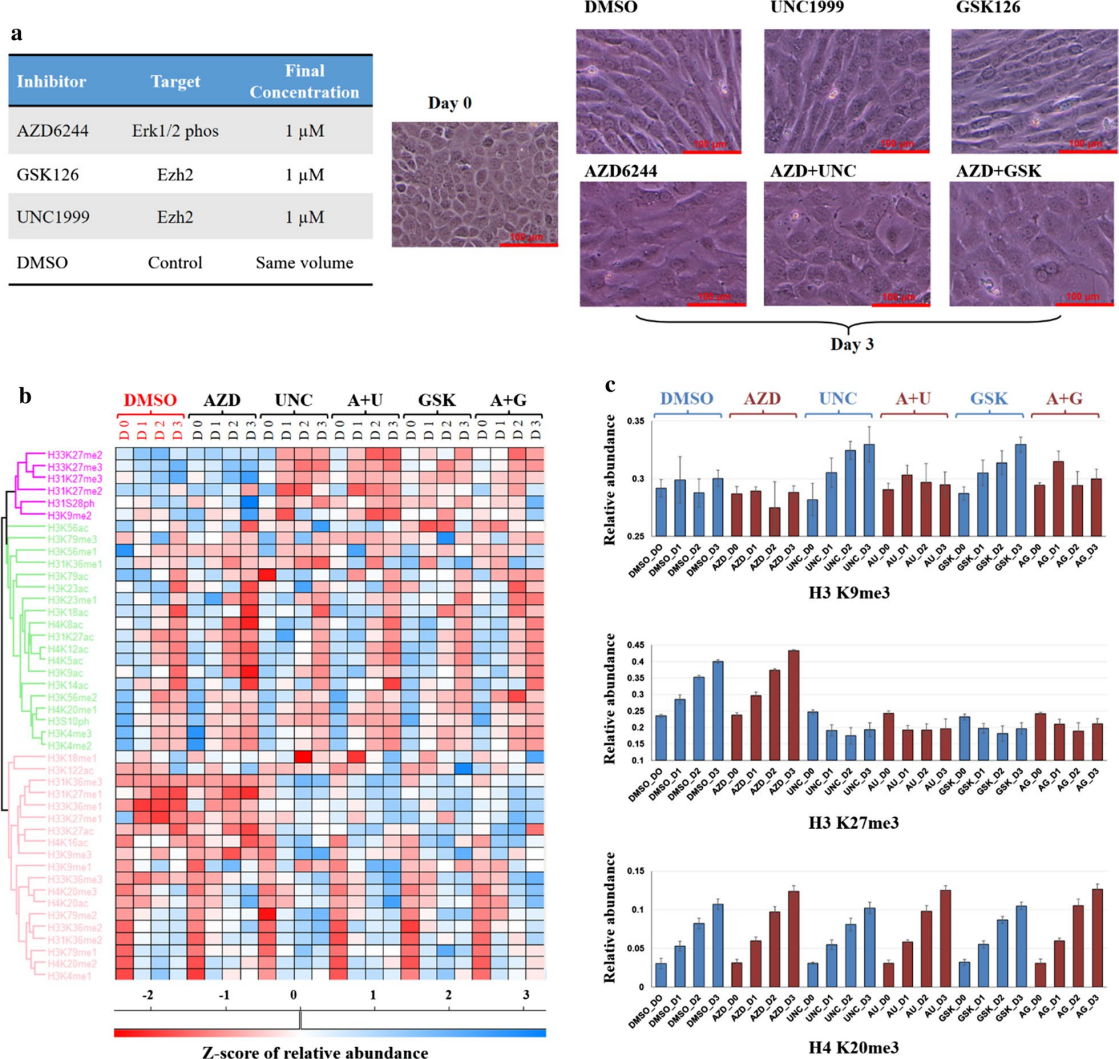
A combination treatment of inhibitors affected the process of EMT. **a** Cell morphology changes after 3-day treatment with different inhibitors. All inhibitors were used with a final concentration of 1 μM. DMSO was used as control with the same volume. Cells were pre-treated with inhibitors for 1 day before adding TGF-β and then treated with TGF-β and inhibitors for another 2 days. **b** Heatmap of z-score of relative abundance of histone H3 and H4 single marks. **c** Relative abundance of selected modified peptides. Error bars represent standard deviation (*n* = 4)

To further explore the possible mechanism, a global epigenetic modifications profiling of samples under specific treatments shown in Fig. 7a was delineated. More than 200 different histone modification types were captured in the dataset (Additional file 1: Figure S9A and Additional file 5: Table S4). Person correlation coefficient (Additional file 1: Figure S9B) and PCA (Additional file 1: Figure S9C) pinpointed the variance and correlation between different samples, where cells under combination inhibitor treatments highly differed from other samples. The distribution of histone H3 and H4 single mark abundance was presented as a heatmap (Additional file 1: Figure S9D), and the expression trends were clustered to group similar histone mark patterns (Fig. 7b).

As expected, H3K27me3 was reduced when Ezh2 inhibitor was present, either alone or together with Erk inhibitor (Fig. 7c). It is interesting to analyze histone modification pattern changes combining with cell morphology changes under different conditions shown in Fig. 7a. Although H3K27me3 does not increase under only Ezh2 inhibitor (UNC1999 or GSK126) treatment, cells still differentiated into mesenchymal phenotype as DMSO control, while the expression of H3K27me3 was not affected when mesenchymal phenotype changes were interrupted by mono Erk inhibitor. As reported, H3K27me3 is essential for EMT [35]. Our histone modification profile also demonstrated that H3K27me3 was significantly increased undergoing EMT (Fig. 6e). Thus, we hypothesized that other histone modifications may be able to function as H3K27me3 when K27me3 could not be expressed and cooperate with transcription factors to trigger EMT. Study has shown close correlations between histone marks associated with gene silencing, such as methylation of H3K9, H3K27 and H4K20 [40]. In fact, we found H3K9me3 to be up-regulated when H3K27me3 decreased under mono Ezh2 inhibitor treatment, while no alteration was found for H4K20me3 between different treatments (Fig. 7c). Therefore, we speculated that the increasing abundance of H3K9me3 might compensate the unchanged H3K27me3, leading to EMT when cells were treated with Ezh2 inhibitors.

Recent study revealed that H3K27me3 depletion caused by EZH2 inhibition can drive to reciprocal H3K27ac gain [14, 41]. In agreement with our data, H31K27ac relative abundance was increased upon 1-day Ezh2 inhibitor treatment, either alone or coexist with Erk inhibitor (Additional file 1: Figure S10). After inducing TGF-β at day 1, H31K27ac was down-regulated in all conditions independent of cell EMT states, implying a more complex cross talk mechanism between signaling pathways and epigenetic regulation leading to EMT phenotypic changes. Regarding other histone modification patterns, e.g., H3K4 and H4K20, no significant changes observed between control and inhibitor treatments (Additional file 1: Figure S10 and Additional file 5: Table S4), which may suggest that these modifications do not play critical roles during EMT.

## Discussion

EMT plays a pivotal role in multiple physiological and pathological conditions, especially regarding cancer progression [3]. Even though the mechanism of EMT signaling is extensively characterized [1], the correlation between signaling pathway and the epigenetic regulation leading to this phenotypic change remains partially obscure. Previous studies investigated only long-term EMT regulation, i.e., from day 1 to 8 [17, 24]. Thus, in order to construct a detail map of signaling information from immediate early to long-term response, we developed and applied a strategy combining triple SILAC for monitoring proteomics time-course changes, TiO_2_ for phosphopeptides enrichment and high accuracy MS characterization for identification of key phosphorylation events in the TGF-β-induced EMT model. The early activation of Erk (in 5-min) was addressed based on phosphosite-kinase specific analysis, indicating the necessity of Erk signaling during EMT. Proteins involved in cell–cell adhesion and cell migration were classified after 1-day TGF-β stimulation. Overall, systematic phosphoproteome and proteome analysis indicates that TGF-β activates first chromatin factors (< 5 min) and then structural proteins involved in cytoskeletal rearrangements (> 1 day).

The comprehensive profiling of histone PTMs identified H3K27me3 as the most significantly up-regulated mark undergoing EMT, demonstrating its critical role in EMT epigenetic regulation which is in accordance with previous reports. Combining phosphoproteomic quantification results, we proposed a mechanism of up-regulation of H3K27me3 expression during EMT by the deactivation of Akt1 and loss of suppression of Cdk1. We then speculated that a combination treatment of Erk and Ezh2 inhibitor can potentiate an enhanced blocking of EMT reprogramming. Subsequently, the hypothesis was supported by visual assessment of cell phonotypic changes under indicated inhibitions. The benefit of the drug combination can be possibly explained by their additive effects on suppressing recruitment of H3K27me3 binding proteins by Ezh2 inhibitor, in addition to inhibiting EMT transcription factor signaling pathway through Erk inhibitor. Moreover, we conjecture that the other gene silencing-related histone mark H3K9me3 may take the role of H3K27me3 and cooperate with signaling pathways to trigger EMT when K27me3 could not be expressed.

## Conclusions

In summary, our data indicate that a proper EMT is achieved only when all necessary component pathways of the regulatory network are activated. Combining histone PTMs analysis with time-resolved quantitative (phospho)proteomics provided a more detailed map of cross talk between signaling pathway and chromatin regulation comparing to previous EMT studies. Our findings point to a promising combination therapy using Erk inhibitor (singling pathway) and Ezh2 inhibitor (epigenetic regulation) to more effectively reduce the progress of aberrant mesenchymal cell proliferation for EMT-related diseases.

## Methods

### Cell culture and stimulation treatments

NMuMG cells were cultured in DMEM media for SILAC (Thermo Fisher Scientific) supplemented with 10% dialyzed fetal bovine serum (dFBS, Gemini Bio-Products) at 37 °C with 5% CO_2_ in atmosphere. Cells were labeled with the natural lysine and arginine or replaced by isotope-labeled amino acids (Silantes, light: lys 0 and arg 0; medium: lys 4 and arg 6; heavy: lys 8 and arg 10) at concentration 100 mg/L for six passengers to make sure fully labeling before adding any treatments. After 1 day 1% dFBS starvation, TGF-β (BioVision, Inc.) was added to induce EMT with a final concentration at 5 ng/ml. Cells were harvested at five time points after stimulation (0 min, 5 min, 60 min, 1 day and 2 days) with triplicates and stored at − 80 °C before further sample preparation for quantification of proteomes and phosphoproteomes.

For histone PTMs analysis undergoing EMT only, un-labeled cells were first cultured in 1% dFBS for 1 day to synchronize cells and harvested at 0 day, 1 day and 2 days. Each condition had three biological replicates. To analyze histone PTMs under inhibitors treatment, cells were pretreated with either individual pERK inhibitor (AZD6244, 1 µM), EZH2 inhibitor (GSK126 or UNC1999, 1 µM), ALK2 inhibitor (LDN-214117, 1 µM), CDK pan-inhibitor (PHA-792887, 0.1 µM), CDK7 inhibitor (BS-181HCl, 1 µM) (Selleckchem) or a combination of two inhibitors after 1-day 1% dFBS synchronization. After 1-day inhibitors pretreatment, TGF-β was added with the presence of inhibitors and cells were cultured for two more days. Epigenetics inhibitors, GSK126 and UNC1999, were added 2 h earlier before adding kinase inhibitors (LDN-214117, AZD6244, PHA-793887, and BS-181HCl) when a combination treatment was performed. Dimethyl sulfoxide (DSMO, Sigma-Aldrich) was used as control. Cell were harvested at 0 day, 1 day, 2 days and 3 days after introducing inhibitors for quantification of histone PTMs. Each condition had four biological replicates. All cell pictures were captured using Microscope at the scale 1:1000.

### Western blot

Cells were harvested at the indicated time points, washed with cold PBS and lysed in RIPA lysis buffer (MilliporeSigma). Western blot (WB) analysis was carried out according to standard protocol. In brief, proteins were separated by NuPAGE 4–12% Bis–Tris Gel (Thermo Fisher Scientific) and electrotransferred to 0.45-μm nitrocellulose membranes (Bio-Rad). After blocking in TBST with 5% BSA (Sigma-Aldrich) at room temperature (RT) for 1 h, the membrane was incubated with primary antibody (1:1000) overnight at 4 °C. Membrane was incubated with HRP-conjugated second antibody (1:10,000) for 1 h at RT after rinsing thrice with TBST. Finally, immunoreactive bands were revealed using ECL Prime Western Blotting Detection Reagent (GE Healthcare Life Sciences) and detected by Amersham Imager 600 (GE Healthcare Life Sciences).

Antibody information: Anti-E-cadherin (Abcam, AB15148), anti-N-cadherin (Abcam, AB18203), anti-Smad2 (CST, 5339T), anti-Smad2 phosphorylation (CST, 3108T), anti-β-tubulin (CST, 2146S) and anti-GAPDH (CST, 5174S).

### Proteomics and phosphoproteomics analysis using nLC-MS/MS

All chemicals used for preparation of nLC-MS/MS sample were of sequencing grade and purchased from Sigma-Aldrich unless otherwise mentioned. Cells were lysed in lysis buffer (6 M urea/2 M thiourea, 50 mM ammonium bicarbonate, pH 8.2, 1*Protease and phosphatase inhibitor cocktail (Thermo Fisher Scientific)), and concentration was measured by Bradford protein assay (Thermo Fisher Scientific). Proteins were mixed as shown in Fig. 1b at the protein ratio of 1:1:1 (L: M: H). Then, samples were reduced by using 5 mM dithiothreitol (DTT) for 60 min at 51 °C and alkylated with 10 mM iodoacetamide (IAA) in dark for 45 min at RT. Proteins were digested first with endopeptidase Lys-C (Wako, MS grade) at an enzyme–substrate ratio of 1:100 for 4 h, after which the solution was diluted four times with 50 mM ammonium bicarbonate. Proteins were then digested with trypsin (Promega) at an enzyme–substrate ratio of approximately 1:50 at room temperature overnight. After digestion, 20 ug proteins were separated out for input proteome analysis, and all left samples were concentrated by SpeedVac for phosphopeptide enrichment.

Titanium dioxide beads (TiO_2_, 5 µm, GL Sciences) were used for selective enrichment of phosphorylated peptides as previously described [42]. Peptide samples were suspended in loading buffer (80% ACN, 5% TFA and 1 M glycolic acid). TiO_2_ beads were subsequently added and incubated at RT for 1 h. Beads were pelleted by centrifugation and the supernatant was discarded. After wash with loading buffer, wash buffer 1 (80% ACN, 1% TFA) and wash buffer 2 (20% ACN, 0.2% TFA), phosphopeptides were eluted 2* with 50 ul elution buffer (40 µl 28% ammonia solution in 980 µl water) and concentrated in SpeedVac.

All samples were stage-tip desalted by C18 (3 M Empore) as previously described [43]. Dried samples were resuspended in buffer A (0.1% (v/v) FA in water) and loaded into a Nano-LC system (EASY-nLC 1000, Thermo Fisher Scientific) coupled online with an Orbitrap Fusion Tribrid mass spectrometer (Thermo Scientific). Peptides were separated on a home-packed capillary column (200 mm length, 75 µm inner diameter) containing reverse-phase ReproSil-Pur C18-AQ resin (3 µm particle size, Dr. Maisch Gmbh) at a flow rate of 300 nl/min. A gradient of 160 min was set from 0 to 38% buffer B (0.1% FA in ACN), then 38–98% buffer B in 10 min. Full scan mass range of m/z 350-1200 was analyzed in the Orbitrap at 120,000 FWHM (200 m/z) resolution and 5.0e5 AGC target value. MS/MS was performed in the ion trap in the normal mode using the TopSpeed mode (3 s) using data-dependent acquisition. HCD collision energy was set to 27, AGC target to 1.0e4 and maximum injection time to 200 ms. Dynamic exclusion (60 s) and apex trigger were enabled. Every sample was injected once into Orbitrap Fusion.

### Quantification of histone PTMs

Histones were acid extracted and propionylated according to standard protocol [31]. Briefly, histones were extracted from nuclei with 0.2 M H_2_SO_4_ and precipitated with 33% TCA (trichloroacetic acid), followed by washes with acetone containing 0.1% HCl and then pure acetone. The resulting pellets were dissolved in 50 mM HEPES, pH8.0. Derivatization reagent was fresh prepared by mixing propionic anhydride with isopropanol at a ratio of 1:3 (v/v), and the mixture was mixed with histone sample at the ratio of 1:4 (v/v) for 15 min at 37 °C. This reaction was performed twice to ensure all free protein N-termini and mono-methylated and free lysine residues were derivatized. Histones were then digested with trypsin (enzyme: sample ratio 1:20) overnight at 37 °C. After digestion, the derivatization reaction was performed twice to react with peptide N-termini.

Samples were desalted using C18 stage-tips before nLC-MS/MS analysis. Chromatography was configured with the same type of column and HPLC as for the proteomics analysis. NanoLC was coupled to a Q-Exactive mass spectrometer (Thermo Scientific). The HPLC gradient was set as follows: 2–28% buffer B in 45 min, 28–80% buffer B in 5 min, 80% buffer B for 10 min at a flow rate of 300 nl/min. A full scan with mass window m/z 300–1100 was acquired in the Orbitrap with a resolution of 120,000 (at 200 m/z), and an AGC target of 5.0e5. MS/MS was performed using a data-independent acquisition mode, and the entire mass range was fragmented at every cycle using windows of 50 m/z. AGC target for MS/MS was 3.0e4, and maximum injection time was 50 ms. HCD collision energy was set at 27. Every condition for no-inhibitor treatment had three biological replicates, and every sample was run thrice by Q-Exactive. Every condition for inhibitors treatment had four biological replicates, and every sample was run only once by Q-Exactive.

### Data processing and analysis

Raw files for proteome and phosphoproteome have been deposited to Chorus database under the accession number 1453.

MS raw files of proteome and phosphoproteome were analyzed by MaxQuant software [44] version v1.6.0.16 against UniProt Mouse protein sequence database (201511). Additionally, the database that included common contaminants was used for first search. Trypsin was specified as digesting enzyme with two missed cleaves allowed. The search for total proteome included fixed modification of carbamidomethyl cysteine and variable modifications of methionine oxidation and N-terminal acetylation. Phosphorylation on serine, threonine and tyrosine residues was also set as variable modification while analyzing phosphoproteome. “Match between runs” was enabled with a matching time window of 1 min. All other values used default setting. Label-free quantification analysis of histone PTMs was performed by home-developed software EpiProfile [9, 33].

ANOVA test was used in the time-course study of proteome and phosphoproteome to identify proteins/PTMs that were significantly changes. Paired sample t-test was performed to identify proteins/PTMs that were significantly changed between two time points. Both phosphoproteome and proteome dynamics were clustered in abundance using fuzzy c-means [21] with at least four out of five values and ANOVA test smaller than 0.05. Gene ontology (GO) enrichment was carried out by using online bioinformatics resource Metascope [45] to characterize the possible association of proteins identified. String (https://string-db.org/) [23] was used to define protein–protein interaction. Site-specific phosphosite-kinase was predicted by iGPS 1.0 [27]. Perseus [46] was used to visualize data from non-supervised hierarchical clustering and principal component analysis plot. Phosphorylation event order was generated by Cytoscape (v3.3.0) [47]. Venn diagram was created by Venn Diagram Plotter (v1.5, https://omics.pnl.gov/). Volcano plots and histograms were built in Excel. Kinome tree was viewed through KinMap [48].

## Additional files

**Additional file 1.** Supplemental information including figures.

**Additional file 2: Table S1.** Time-course monitoring of protein abundance changes during EMT. (A) Total list of identified and quantified proteins across five time points. Protein IDs refer to UniProt database. Peptide counts highlight the number of razor and unique peptides used for protein quantification. Protein intensity is the raw intensity, and the normalized log_2_ transformed protein abundance (average of biological replicates, *n* = 3). 0 min_S and 0 min_L refer to Fig. 1b. ANOVA *p* value describes the ANOVA *p* value of protein levels. Cluster number refers to Figure S4A (blank means protein not used for clustering); isClusterMember determines whether the protein belongs significantly to the cluster assigned. (B) Annotation for cluster proteins. (C) GO-BP (gene ontology biological processes) enrichment results for cluster proteins.

**Additional file 3: Table S2.** Time-course monitoring of phosphorylation site abundance changes during EMT. (A) Total list of identified and quantified phosphosites across five time points. Protein IDs refer to UniProt database. Mod site highlights the phosphorylated S/T or Y residue within protein amino acid (aa) sequence. Localization prob is the confidence score for site localization of the phosphorylation (1 means fully unambiguous). Sequence window highlights the 31 aa residue within protein. Phosphosite intensity is the raw intensity and the normalized log2 transformed phosphosite abundance adjusted based on protein abundance (average of biological replicates, *n* = 3). 0 min_S and 0 min_L refer to Fig. 1b. ANOVA *p* value describes the ANOVA *p* value of phosphorylation levels. Cluster number refers to Figure S4A (blank means the indicated phosphosite was not used for clustering); isClusterMember determines whether the protein belongs significantly to the cluster assigned; ‘is Kinase’ determines whether the protein belongs to kinome; ‘is related with histone PTMs’ determines whether the protein has been described with histone PTMs modifiers. (B) Kinases predicted by iGPS which are responsible for significantly regulated (*p* value < 0.05 based on triplicates) phosphopeptides at the indicated times after TGF-β stimulation. (C) Annotation for cluster phosphoproteins. (C) GO-BP (gene ontology biological processes) enrichment results for cluster phosphoproteins.

**Additional file 4: Table S3.** Quantification of histone modifications during EMT. (A) Relative abundance of histone peptides detected across three time points. (B) Deconvoluted single marks from table A. For simplicity, the relative abundance of single PTMs was extracted by summing all peptides carrying the given mark. Each day has three biological replicates, and each sample has three instrument replicates. For example, D0_1_2 means the second injection of first biological sample for Day 0.

**Additional file 5: Table S4.** Quantification of histone modifications under different inhibitors treatment. (A) Relative abundance of histone peptides detected across three time points. (B) Deconvoluted single marks from table A. For simplicity, the relative abundance of single PTMs was extracted by summing all peptides carrying the given mark. Each treatment has four biological replicates. For example, D1_D0 means DMSO control for Day 0. A1_D1 indicates AZD6244 treatment at Day 1. U: UNC1999; AU: AZD6244 plus UNC1999; G: GSK126; AG: AZD6244 plus GSK126.

## Abbreviations

ACN: acetonitrile
dFBS: dialyzed fetal bovine serum
DIA: data-independent acquisition
DMSO: dimethyl sulfoxide
DTT: dithiothreitol
EMT: epithelial to mesenchymal transition
FA: formic acid
HCl: hydrochloric acid
H_2_SO_4_: sulfuric acid
IAA: iodoacetamide
GO-BP: gene ontology biological processes
MS: mass spectrometry
PCA: principal component analysis
PTM: post-translational modification
RT: room temperature
SILAC: stable isotope labeling by amino acids in cell culture
TCA: trichloroacetic acid
TFA: trifluoroacetic acid
TGF-β: transforming growth factor beta
WB: western blotting
