# Supplementary material for: Coordination between TGF-β cellular signaling and epigenetic regulation during epithelial to mesenchymal transition
Source: Epigenetics Chromatin. 2019 Feb 8;12:11. doi: 10.1186/s13072-019-0256-y (PMC6368739; doi:10.1186/s13072-019-0256-y)

**Coordination between TGF-β cellular signaling and epigenetic regulation during epithelial to mesenchymal transition**

Congcong Lu ^1^, Simone Sidoli ^1^, Katarzyna Kulej ^1, 2^, Karen Ross ^3^, Cathy H. Wu ^3^, Benjamin A. Garcia ^1, *^

^1^Epigenetics Institute, Department of Biochemistry and Biophysics, Perelman School of Medicine, University of Pennsylvania, Philadelphia, PA 19104, USA

^2^Division of Cancer Pathobiology, Children's Hospital of Philadelphia, Philadelphia, PA 19104, USA

^3^Center for Bioinformatics & Computational Biology, Department of Computer & Information Sciences, University of Delaware, Newark, DE 19711, USA

*Correspondence: [bgarci@pennmedicine.upenn.edu](mailto:bgarci@pennmedicine.upenn.edu)

**SUPPLEMENTAL INFORMATION**

**SUPPLEMENTAL FIGURE LEGENDS**

**Figure S1.** Scatter plots showing the correlation of WB profiles and corresponding quantitative MS data for individual protein based on the average of log_2_ fold change shown in Figure 2B. The linear correlation coefficients (R2) are indicated in each plot.

**Figure S2.** Boxplots representing proteome and phosphoproteome data distribution before and after normalization. The bottom figure shows that the normalization was effective, as all data became equally centered. L: K0R0, M: K4R6, H: K8R10. B1, B2 and B3 were bio-replicate 1, 2 and 3. Details for Exp. 1 and Exp. 2 can be found in Figure 1B.

**Figure S3.** Quantitative and qualitative analysis of proteome and phosphoproteome. (A) Total number of quantified proteins and phosphosites at indicated stimulation times. 0 min_L indicates the sample of 0 min from Exp. 1, and 0 min_S from Exp. 2. Detailed experiments information can be found in Figure 1B. (B) Venn diagram showing the overlap between identified proteins and phosphoproteins. (C) Histogram showing the TiO_2_ enrichment efficiency for each experiment. (D) Pie chart showing the distribution of the number of identified phosphosites with different localization probabilities at single amino acid level.

**Figure S4.** Clustering of dynamic phosphoproteome and proteome profiles. (A) Cluster profiles for proteome and phosphoproteome. At least 4 valid numbers out of 5 time points and ANOVA test smaller than 0.05 were clustered in abundance using fuzzy c-means. (B) Numbers of proteins, phosphosites and phosphoproteins presented in each cluster. (C) Heatmap showing different GO biological processes enrichment for each clusters. Grey means missing value.

**Figure S5.** Protein-protein interaction of phosphoproteins classified in cluster 1 and Cluster 2, respectively.

**Figure S6.** Kinome tree view of identified phosphorylated kinases (labeled in red).

**Figure S7.** Profiling of histone modifications changes during EMT. (A) Heatmap of histone H3 and H4 single marks relative abundances at different TGF-β stimulation time with triplicates. (B) Heatmap of z-score of relative histone H3 and H4 single marks abundances at different TGF-β stimulation time with triplicates.

**Figure S8.** Cell morphology changes under different inhibitors’ treatments. (A) Cell morphologies presented at day 3 under different inhibitors’ treatments. (B) Detailed day by day cell morphology changes for specific treatments. Cells were pre-treatment with inhibitors for 1 day before inducing two more days TGF-β stimulation with the attendance of inhibitors.

**Figure S9.** Profiling of histone modifications changes after inhibitors treatment. (A) Number of identified histone peptides carrying different type of modifications at each condition. (B) Person correlation coefficient analysis of histone modifications under different treatments. (C) PCA clustering of histone modifications based different inhibitor treatments or different treatment times. The data supplied to generate PCA were all detected histone peptides provided in Table S4A, including modified and unmodified peptides. (D) Heatmap of histone H3 and H4 single marks abundances at different conditions.

**Figure S10.** Relative abundance of selected modified peptides after inhibitors treatment. Error bars represent standard deviation (n=4).

**SUPPLEMENTAL FIGURES**

**Figure S1.**


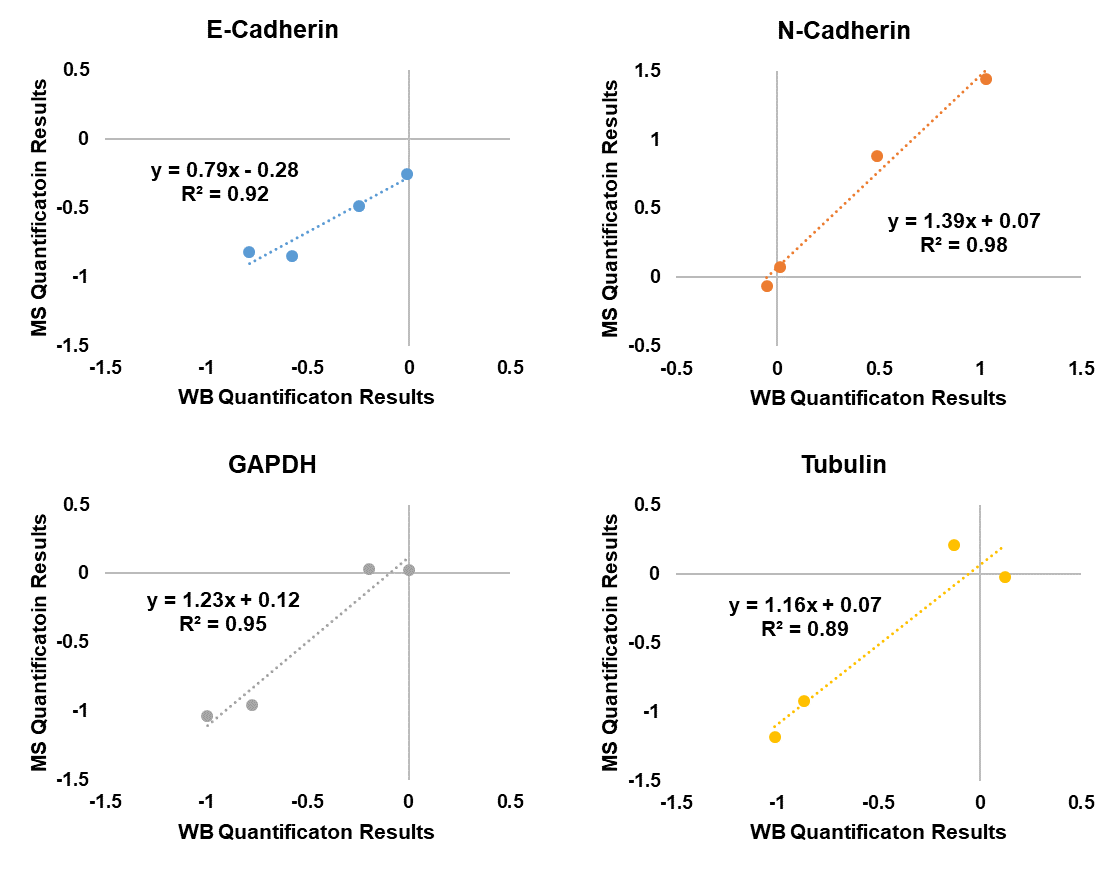


**Figure S2.**


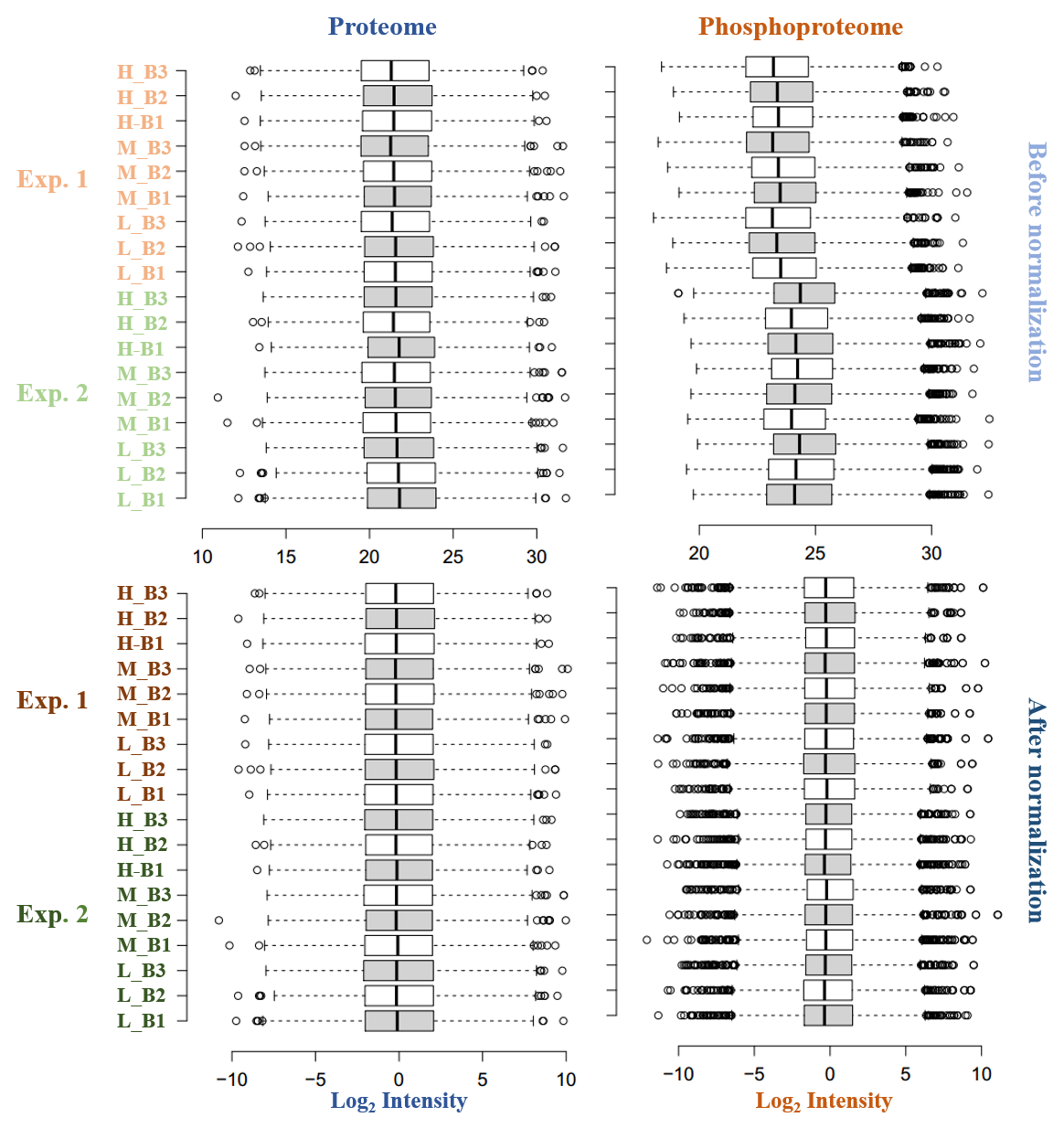


**Figure S3.**


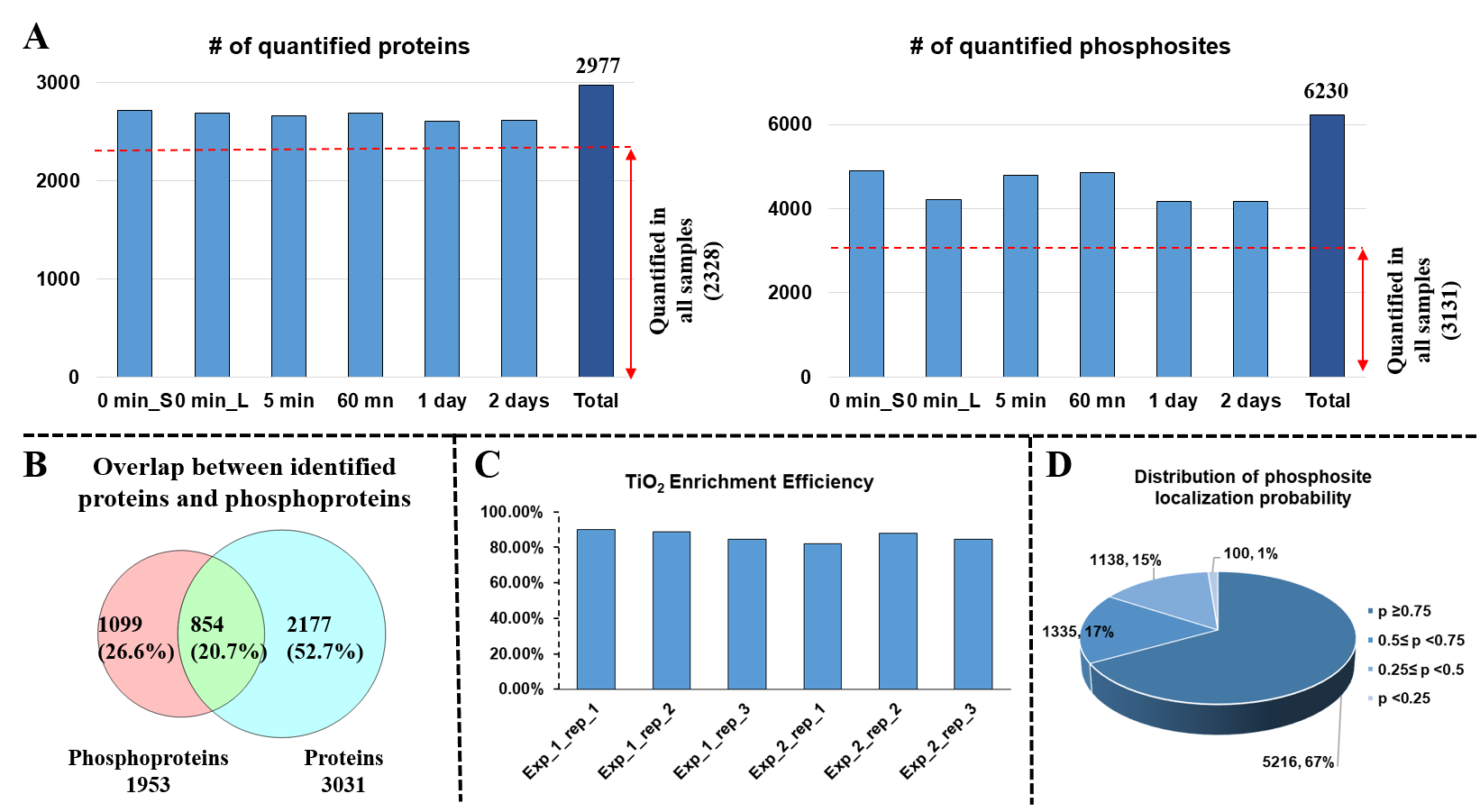


**Figure S4.**


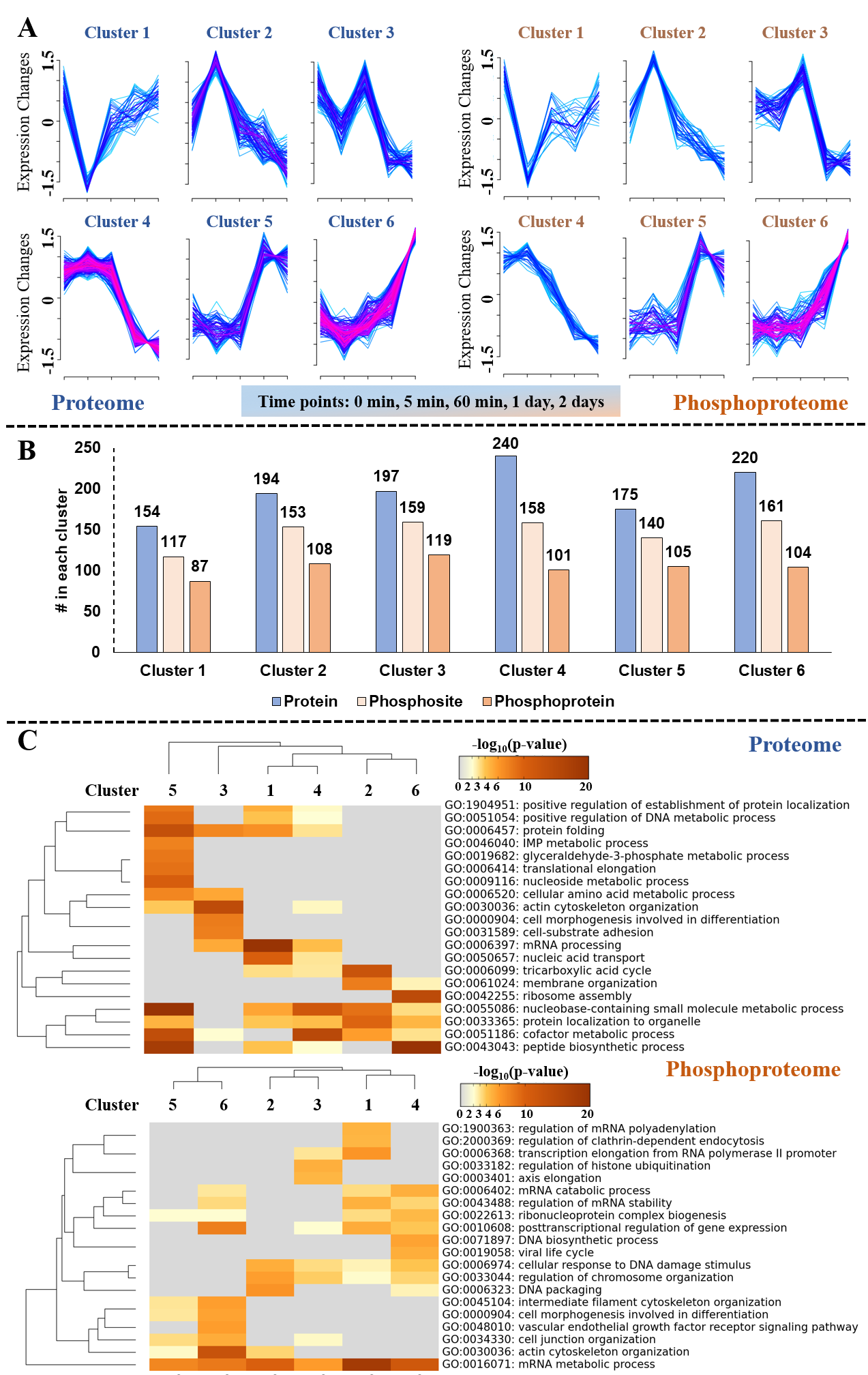


**Figure S5.**


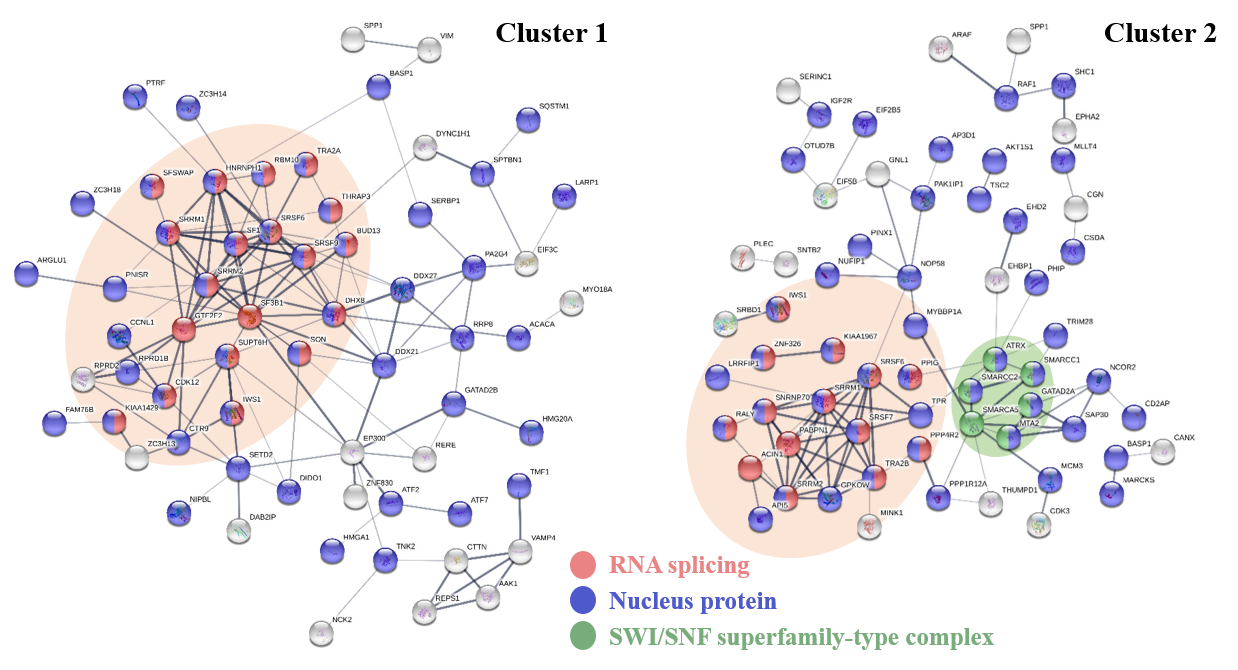


**Figure S6.**


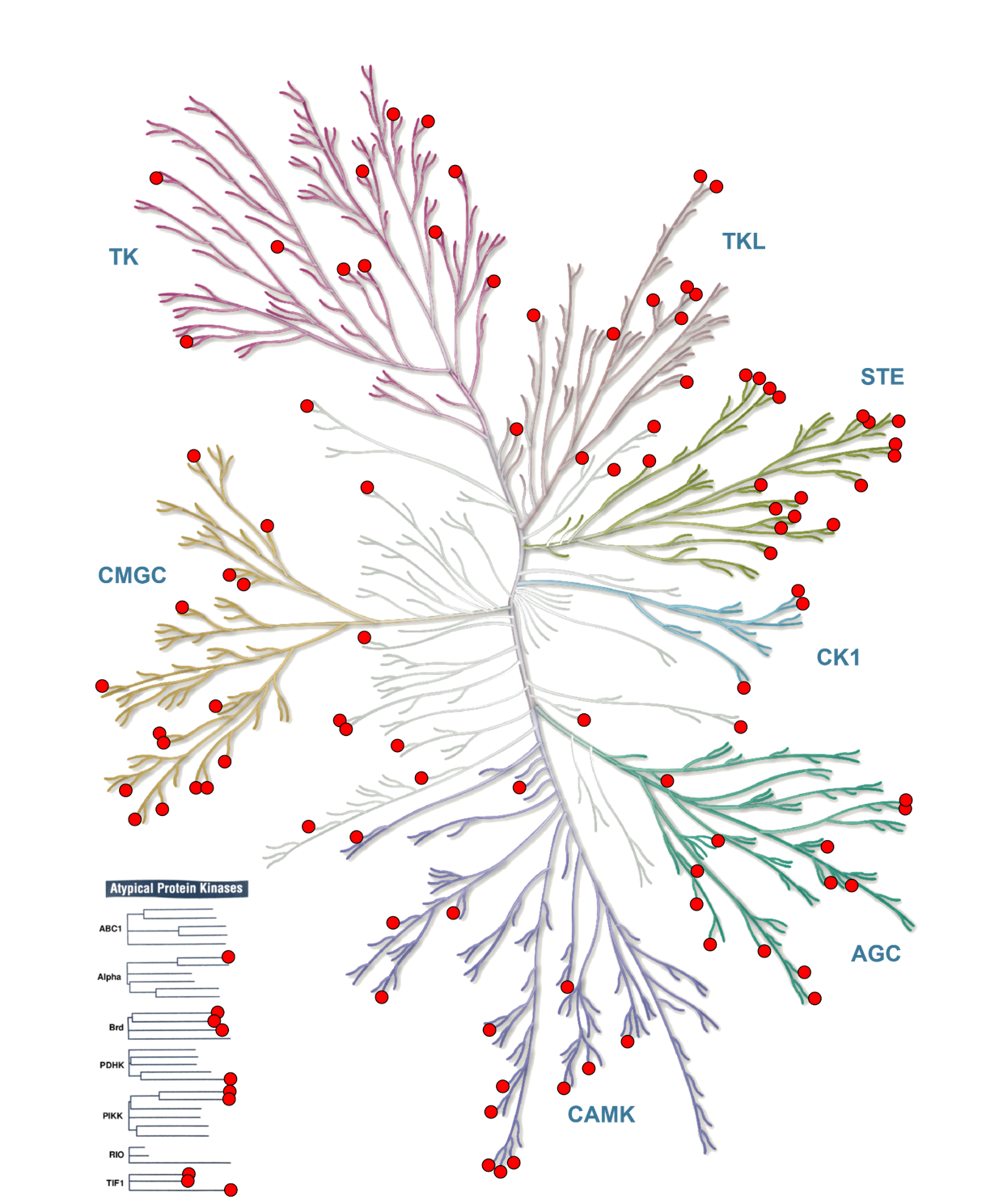


**Figure S7.**


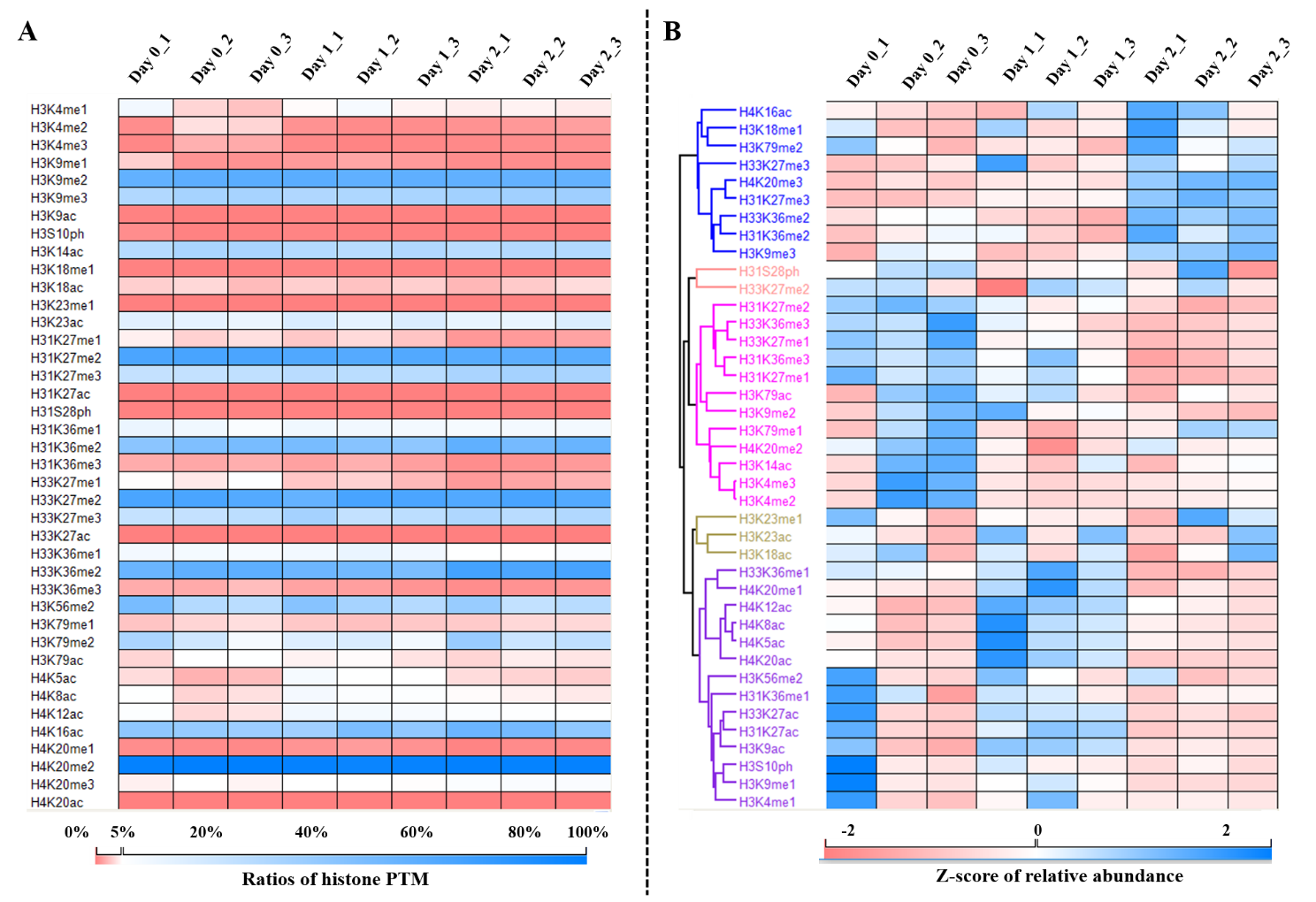


**Figure S8.**


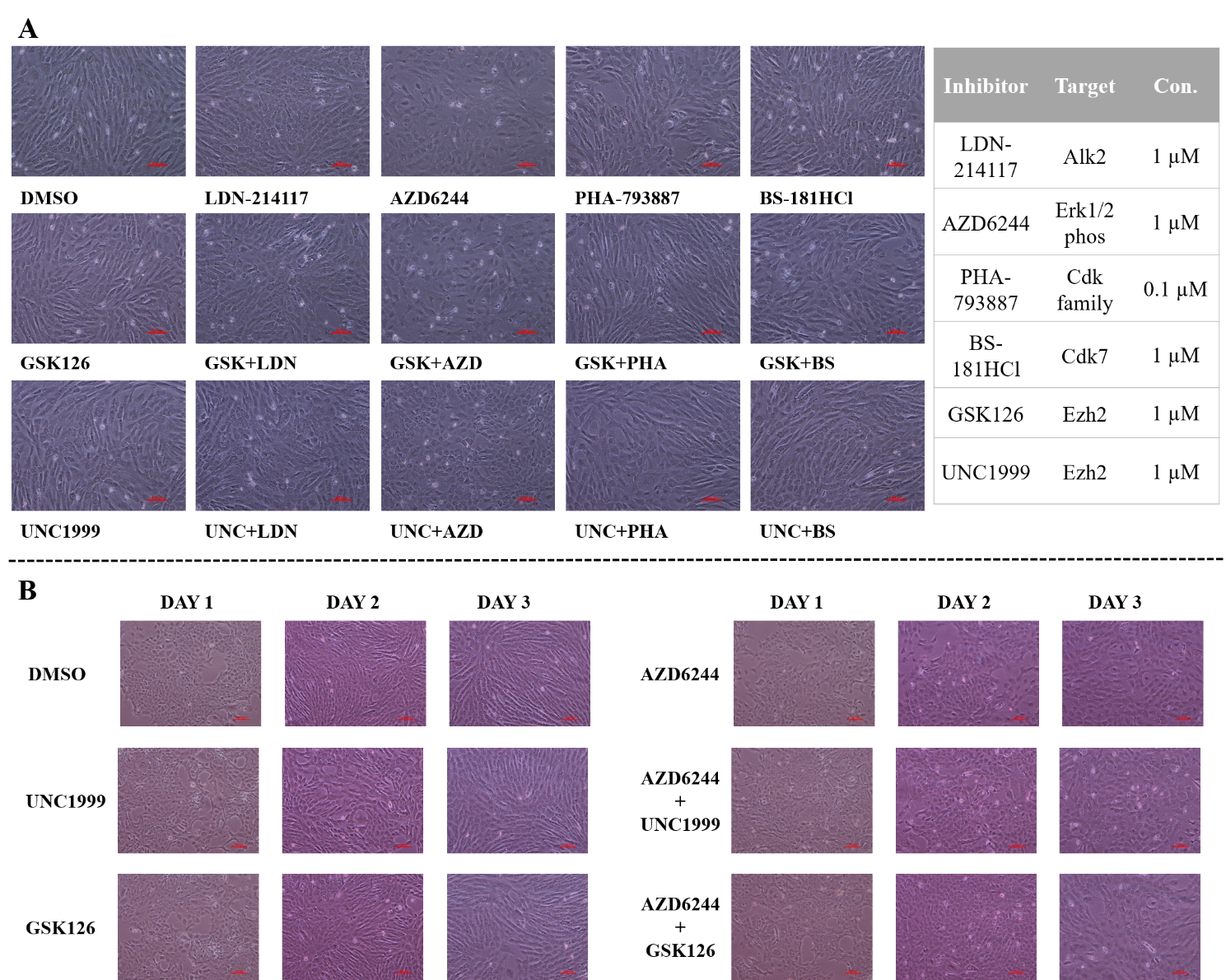


**Figure S9.**


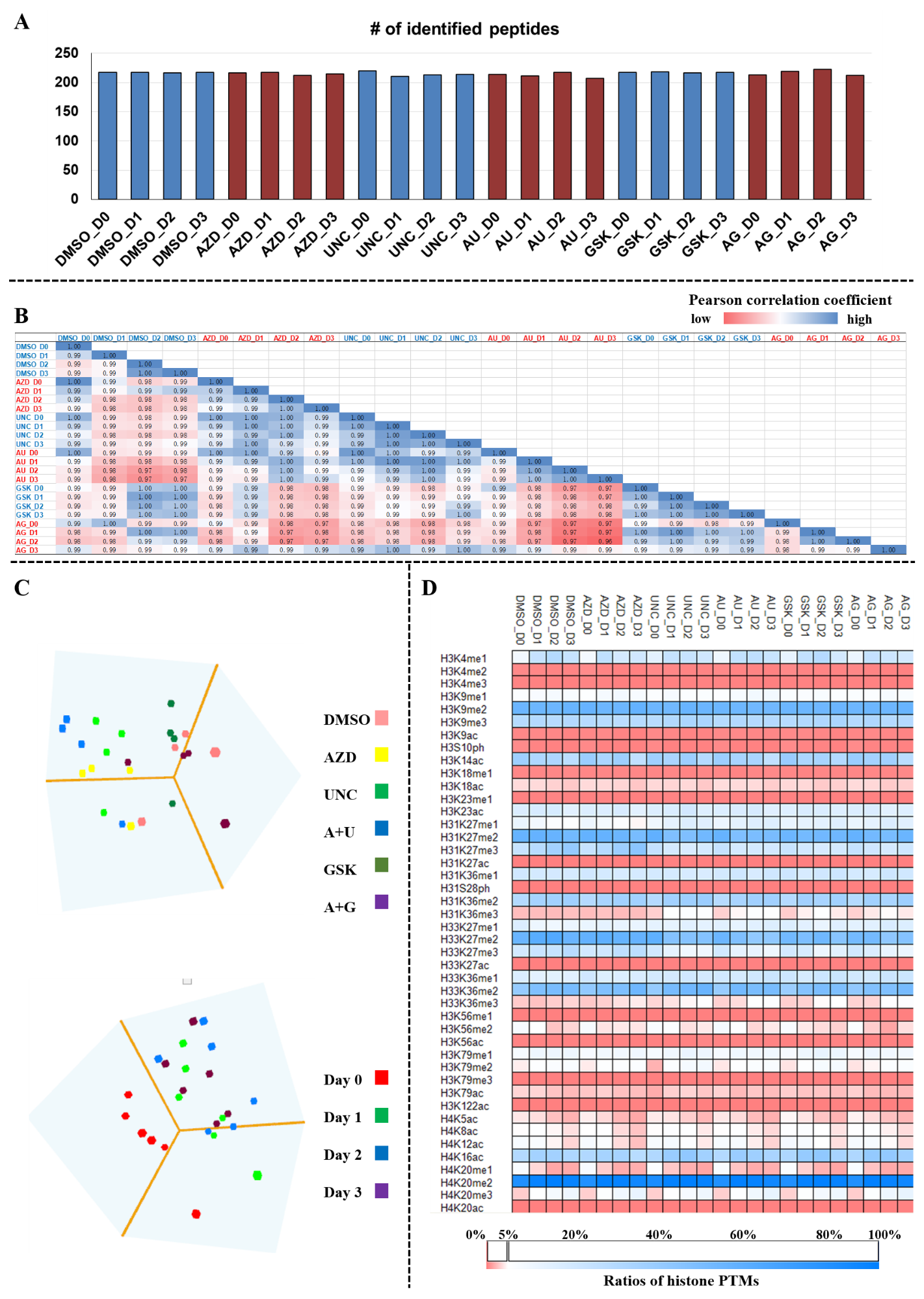


**Figure S10.**


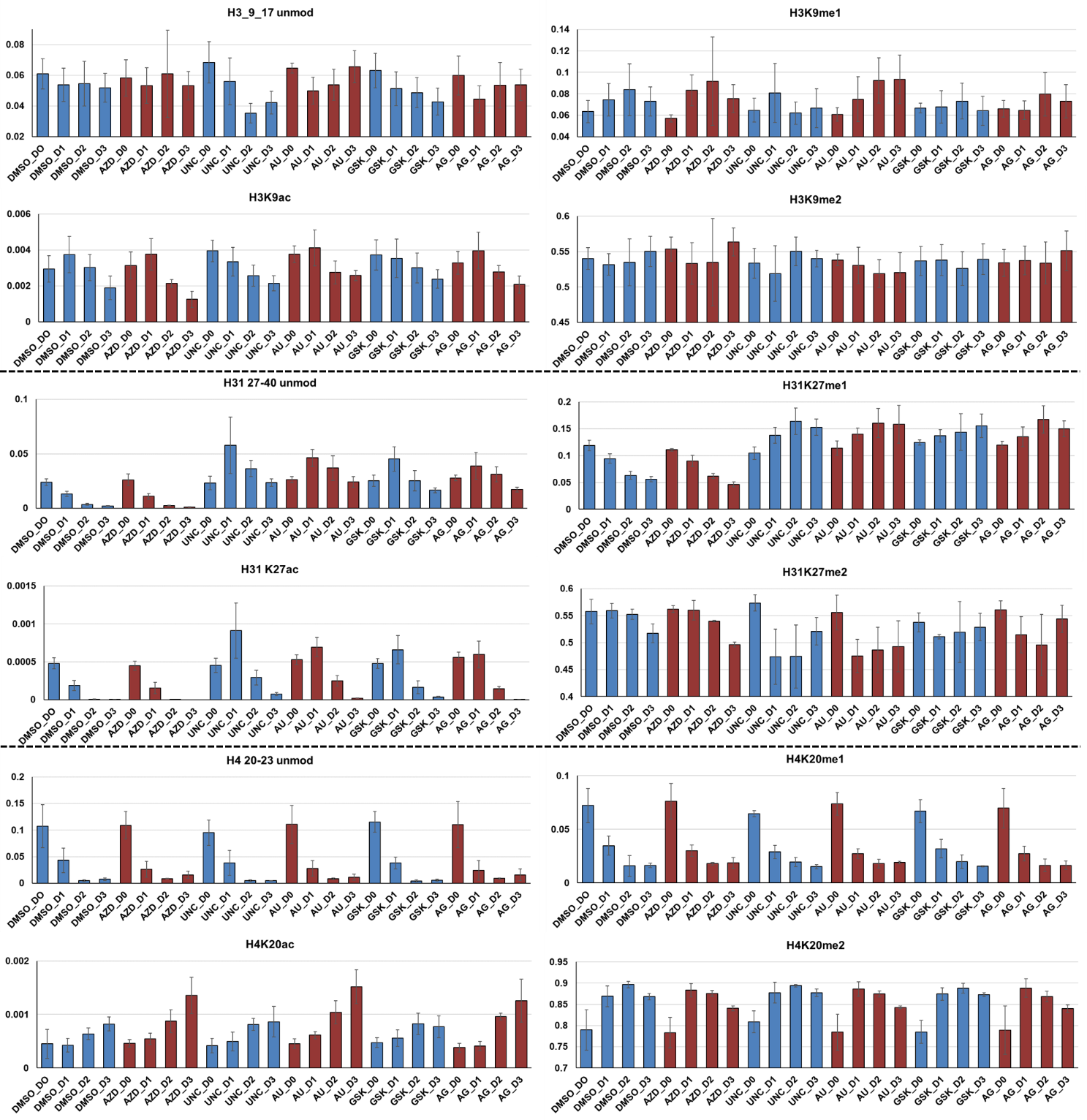

Supplement: Supplementary file 1 — Additional file 1. Supplemental information including figures. [file 13072_2019_256_MOESM1_ESM.docx]
